# Biomedical Porous Shape Memory Alloys for Hard-Tissue Replacement Materials

**DOI:** 10.3390/ma11091716

**Published:** 2018-09-13

**Authors:** Bin Yuan, Min Zhu, Chi Yuen Chung

**Affiliations:** 1 School of Materials Science and Engineering, South China University of Technology, Guangzhou 510640, China; apsheng@scut.edu.cn; 2 Key Laboratory of Advanced Energy Storage Materials of Guangdong Province, Guangzhou 510640, China; 3 Department of Physics & Materials Science, City University of Hong Kong, Kowloon, Hong Kong, China; appchung@cityu.edu.hk

**Keywords:** shape memory alloy, NiTi, β type Ni-free Ti alloy, porous material, surface modification, biocompatibility

## Abstract

Porous shape memory alloys (SMAs), including NiTi and Ni-free Ti-based alloys, are unusual materials for hard-tissue replacements because of their unique superelasticity (SE), good biocompatibility, and low elastic modulus. However, the Ni ion releasing for porous NiTi SMAs in physiological conditions and relatively low SE for porous Ni-free SMAs have delayed their clinic applications as implantable materials. The present article reviews recent research progresses on porous NiTi and Ni-free SMAs for hard-tissue replacements, focusing on two specific topics: (i) synthesis of porous SMAs with optimal porous structure, microstructure, mechanical, and biological properties; and, (ii) surface modifications that are designed to create bio-inert or bio-active surfaces with low Ni releasing and high biocompatibility for porous NiTi SMAs. With the advances of preparation technique, the porous SMAs can be tailored to satisfied porous structure with porosity ranging from 30% to 85% and different pore sizes. In addition, they can exhibit an elastic modulus of 0.4–15 GPa and SE of more than 2.5%, as well as good cell and tissue biocompatibility. As a result, porous SMAs had already been used in maxillofacial repairing, teeth root replacement, and cervical and lumbar vertebral implantation. Based on current research progresses, possible future directions are discussed for “property-pore structure” relationship and surface modification investigations, which could lead to optimized porous biomedical SMAs. We believe that porous SMAs with optimal porous structure and a bioactive surface layer are the most competitive candidate for short-term and long-term hard-tissue replacement materials.

## 1. Introduction

The average life-span of modern person has been greatly elongated by the advanced medical technology and living condition. Thus, the population of aged people (60 years old or above) has increased tremendously in recent decades all around the world, especially in the United States (US), Europe, Japan, and China. It had been reported that the population of older persons is 962 million, which is 13% of the world population in 2017, and it is estimated to reach 2.1 billion in 2050 [1]. It is well known that 90% of the aged population, even including those people of age 40–59, easily suffers from all kinds of degenerative diseases, such as osteoporosis or arthritis [2]. Until now, the best solution of curing these degenerative diseases is surgical restoration or replacement while using artificial biomaterials, such as replacement implants in hips, knees, spine, shoulders, and dental structures [3,4]. Thus, there is a tremendous demand in those artificial biomaterials, especially, the long-term implantable biomaterials due to longer life expectancy or the surgeries in young men.

**Figure 1:**
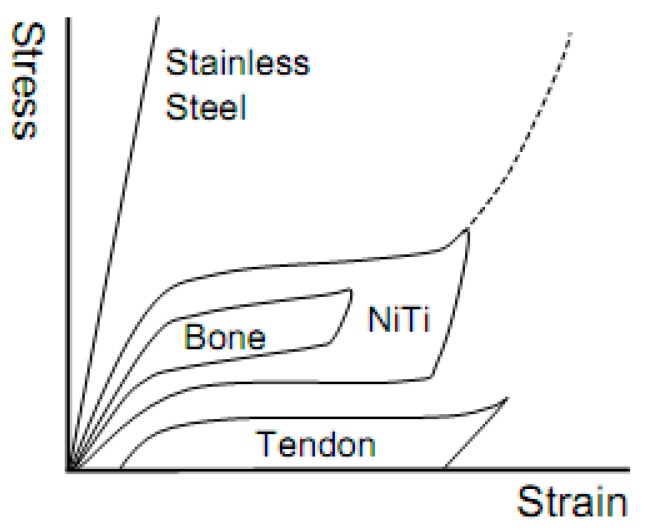
The stress-strain curves for dense NiTi shape memory alloys (SMAs), stainless steel, bone, and tendon [6].

Among those current hard-tissue implantable biomaterials, commercially pure titanium or titanium alloys are considered as the best choice because of their good load-bearing capability, high specific strength, excellent corrosion resistance, and biocompatibility [5]. Moreover, a kind of novel Ti alloys (Shape Memory Alloys, SMAs) with unique shape memory effect (SME) or superelasticity (SE), including NiTi and β type Ni-free Ti-based SMAs, have been attracted more attentions in recent half a century. The unique SE of SMAs near ambient temperature is similar to the mechanical behaviors of some hard-tissues, such as human bones or tendons, as shown in Figure 1 [6]. In addition to SE and SME, SMAs exhibit excellent mechanical properties, including high strength and fatigue life, as well as a relatively low elastic modulus [5]. All of these make SMAs become a kind of superior alloys for various hard-tissue implantations, including dental devices, joint replacements, spine fracture fixations, etc. [6,7].

The SME was first found in an Au-Cd alloy in 1951 by Chang and Read [8], and then, in Cu-Zn, In-Tl, and Cu-Al-Ni alloys. However, it was until the discovery of SME in equiatomic Ni-Ti alloys by Buehler et al. in 1963 [9], SMAs have attracted great attentions as smart materials and applied in actuators, sensors, etc. Since the early 1980s, NiTi SMAs have been introduced to medicine and dentistry due to their excellent SME, SE, mechanical stability, corrosion resistance, and biocompatibility. They have been widely recognized and accepted for medical applications in dentistry and orthopaedics. Moreover, the excellent malleability and ductility of NiTi SMAs allow for them to be manufactured in the forms of wires, tubes, or sheets, and providing a wide spectrum of vascular interventions applications. Thus, biomedical applications have already become one of the important targets nowadays [6].

However, the high modulus of bulk NiTi SMAs when comparing with human bones can lead to some problems, such as local osteoporosis, in the orthopaedic implantation application, because the load can not be transferred from the implants to the adjacent bone tissues, it called “stress shielding” phenomenon which leads to bone resorption around the implants. Thus, porous NiTi SMAs become the research focus of hard-tissue implantable or replacement materials because of their low modulus and additional benefits, such as lowering and matching elastic modulus with various hard-tissues, promoting cells adhesion and in-growth, and allowing for body fluid exchanging [10]. Many works [11,12,13,14,15] have been dedicated to the fabrication, mechanical, and biological performances of porous SMAs. For example, porous NiTi SMAs have been fabricated successfully by various methods already, such as vacuum sintering, self-propagating high temperature synthesis (SHS), and capsule-free hot isostatic pressing (CF-HIP). Porous NiTi SMAs with 30% porosity can reach as high as 4% SE at room temperature (RT), moreover, they exhibit better biocompatibility than the dense forms. The porous NiTi SMAs have shown great advantages in biomedical applications [10,14].

In the present article, we review recent progress on the mechanical and biological properties of porous SMAs in relation to their porous structures and microstructures in three sections. It covers (1) the brief description on the fundamentals of SME and SE; (2) the fabrication methods and pore structure controlling, mechanical and biological performances of porous NiTi SMAs in relation to their porous structure and microstructure, surface modification, and biomedical applications; and, (3) the mechanical and biological properties of dense and porous Ni-free Ti-based SMAs. Finally, this review summarizes past works and gives a future prospect. It should be mentioned that several review articles have been published with an emphasis on different aspects of the porous NiTi or Ti-based SMAs [10,16,17]. The reader can refer to those reviews for more information.

## 2. Fundamentals of Shape Memory Alloys (SMAs)

**Figure 2:**
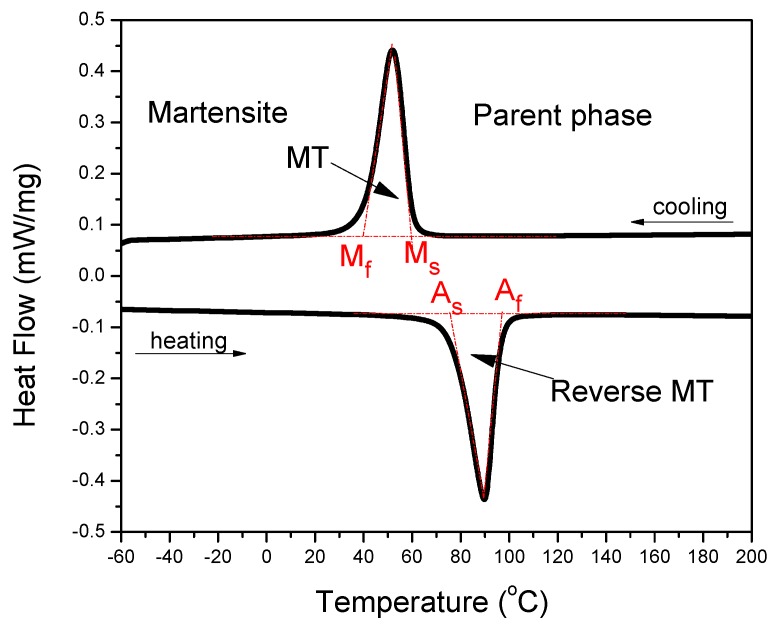
Schematic determination of MT temperature in a NiTi SMA via a differential scanning calorimetry (DSC) plot [19].

SMAs are one kind of alloys, which can exhibit SME originated from the thermoelastic martensitic transformation (MT), which is an important displacive solid phase transformation and it has been widely studied in the steels, non-ferrous alloys, and ceramics for its great influence on their properties [18]. Several behaviors that are related to SME may be presented in the SMAs, which undergo different thermo-mechanical treatments or external conditions, including one-way SME, two-way SME, all-round SME, rubber-like behavior, and magnetic SME [17]. One-way that SME is the most frequently utilized behavior in various applications, especially in medical applications. It refers to the phenomenon that an alloy is deformed in its cold state (i.e., in martensite phase, below Ms temperature, as shown in Figure 2) and holds its shape, and then it would recover its original shape upon heating to above a transition temperature of A_f_ temperature, as shown in Figure 2 (now in the high-temperature state, called parent phase). That means the SMA can remember its original shape. The MT temperature can be directly determined through many techniques, including differential scanning calorimetry (DSC) and electrical resistivity measurement as a function of temperature. Figure 2 presents the determination of MT temperature in a NiTi SMA via a DSC curve through the typically tangential method [19].

The SME can be simply understood from the crystallographic reversibility of MT. As well known, MT is a first order phase transformation, a certain strain arises around the martensite when it is formed from parent phase due to the lattice volume difference between martensite and parent phase. When the strain is too big to be released elastically, high density of defects would produce in both martensite and parent phase, and make the interface unmovable. However, the strain is small in thermoelastic MT and it can be accommodated by forming a self-accommodated martensite. That is, the parent phase with high symmetry transforms to several “martensite variants” and each variant has a unique but crystallographically equivalent orientation relationship to the parent phase [17,18]. The number of variants is only determined by the symmetry of the parent phase and the martensite. In this situation, the strain from one variant counteracts by another and the martensite has minimum strain energy, and there is no distinct macroscopic shape change after phase transformation.

**Figure 3:**
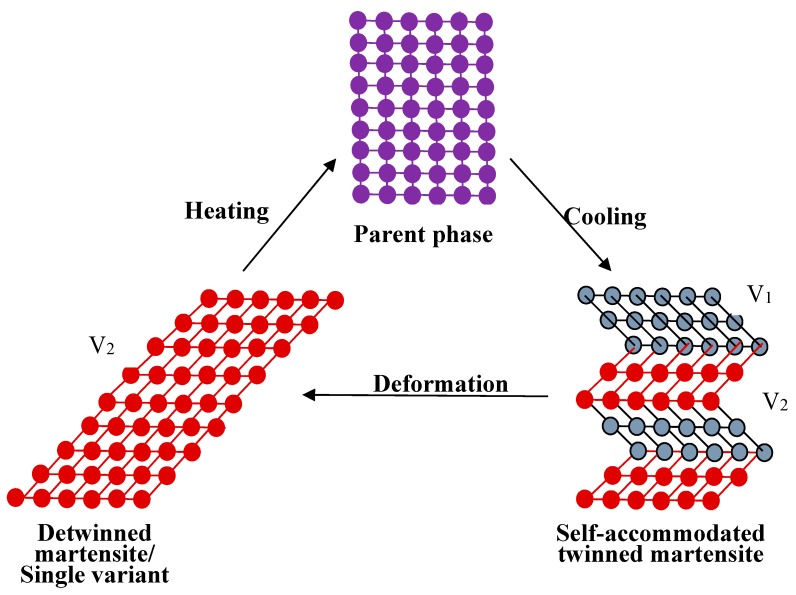
Two-dimensional (2D) crystallographic mechanism of SME (V_1_ represents one martensite variant, V_2_ represents another variant).

**Figure 4:**
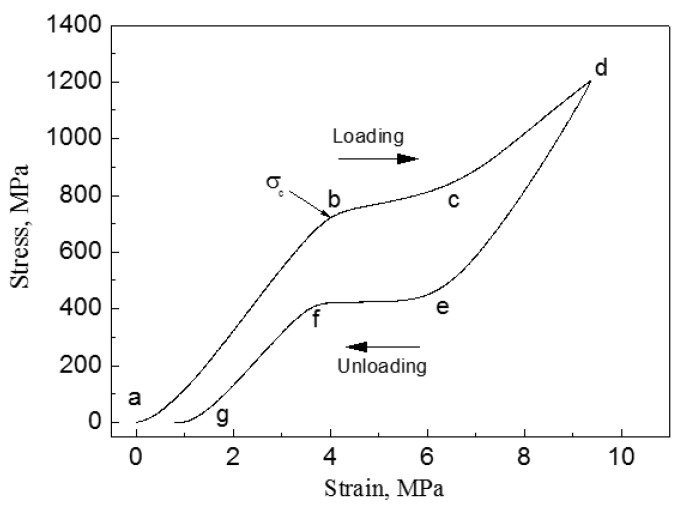
Stress-strain curve of superelasticity (SE) in an SMA (ab—elastic deformation of austenite, bc—forming martensite by stress induced martensitic transformation (SIMT), cd—elastic deformation of martensite, de-recovery of elastic deformation of martensite, ef—austenite forming by reverse MT, fg—recovery of elastic deformation of austenite).

When an external stress applies to an SMA with self-accommodated martensite, the variant with strain direction most favorable to that of the applied stress would swallow up other variants. The macroscopic shape change would appear because the strain that is produced by those variants with different orientations does not counteract each other but accumulate. This is called the reorientation of martensite variants [17]. The shape change would not disappear even upon unloading. However, when the reorientated martensite variant transforms back to the parent phase by heating, it should follow the lattice correspondence relationship of the parent phase transform into the variant. Thus, the shape change would be recovered after the variant transforms back to the original parent phase. Figure 3 illustrates schematically the two-dimensional (2D) crystallographic mechanism of SME. SE, or called as pseudoelasticity (PE), is another unique behavior of SMAs, which is directly related to stress induced martensitic transformation (SIMT) in SMAs, and the mechanism of SE is similar to the SME. It refers to the ability of SMAs to recover a large nonlinear deformation (even reach as high as ~18% in some SMAs [20]) immediately upon unloading at a constant temperature, and one typical stress-strain curve of SE is shown in Figure 4. In fact, the external stress alters the thermodynamic conditions of MT, according to the shear-like mechanism and thermodynamic principle [17]. Both SME and SE are greatly affected by many factors, including inherent microstructural features and the external conditions, such as applied stress and temperature [21]. In general, SME occurs by deforming below A_s_ and following heating above A_f_, while SE occurs above A_f_ depending upon the applied stress.

## 3. Requirements for Hard-Tissue Replacements

The perfect method to repair/regenerate hard tissues is to use autografts, which the tissue that is taken from one site and grafted to another site on the same person. However, a large number of artificial biomaterials have to be adopted in clinic therapy surgeries due to the limitations on the availability of natural hard-tissue. The ideal biomaterials should possess the same properties/functionality and structure with human hard-tissue as far as possible. Therefore, we briefly describe the structure and properties of these human hard tissues.

**Figure 5:**
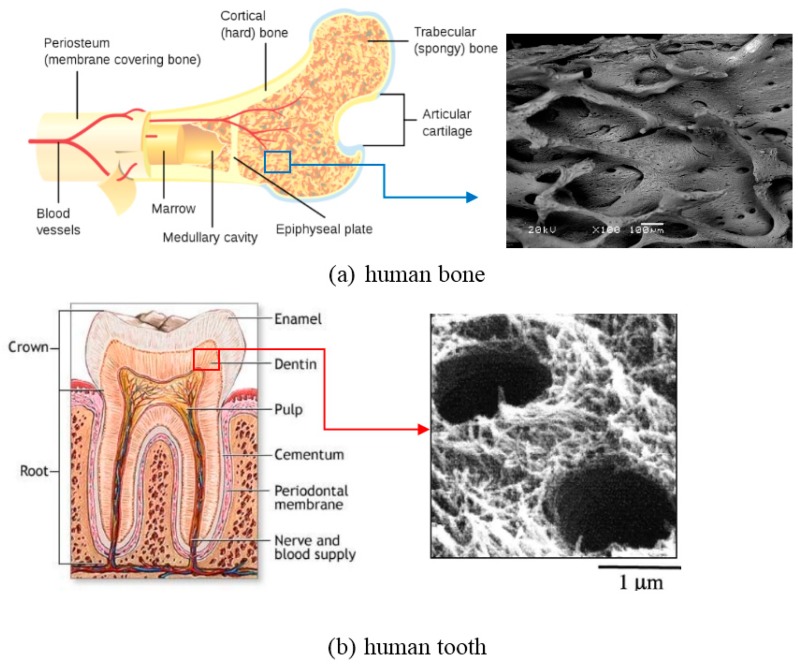
Microstructure illustration of human hard-tissue: (**a**) human bone [23] and (**b**) human tooth [34] (magnification image is SEM of actual tissue in a specific region).

The hard-tissue in the human body mainly refers to two categories, bone and tooth. The human bone mainly consists of outer cortical bone (also called compact bone, relatively dense) and inner cancellous bone (also called trabecular bone, porous or cellular), except some vascular tissues [22,23], as shown in Figure 5a. Both two types of bone tissues are in form of hydroxyapatite (HA) when removing the organic matter, and differ only in porosity and density. The porosity of cortical bone is 5–30% [23], while the porosity ranges from 30% to 90% in cancellous bone [22]. Although the distinction between the two bone tissues is fairly difficult for the porosity less than 50%, the change from the cortical to the cancellous bone is usually clear and takes place over a small distance, i.e., gradient porosity from outside to inside. The physical and mechanical properties of these two kinds of bones from the reported results [22,23,24,25,26,27,28,29,30,31,32] are listed in Table 1. The human teeth are composed of external enamel (relatively compact) and internal dentin (porous or tubular layer), in which the enamel has a high degree of mineralization in form of HA, and dentin composed of type-I collagen, fluid, and nanocrystalline HA, as shown in Figure 5b. The physical and mechanical properties of human teeth are also listed in Table 1 [22,23,24,25,26,27,28,29,30,31,32,33,34,35].

Although the concrete requirements for hard-tissue replacements depend on the specific applications in different parts or positions, the following general requirements should be considered for hard-tissue replacement materials:

**(1) Satisfactory biocompatibility and corrosion resistance**

First, the materials that are used as implantable parts should be bio-inert and highly non-toxic at least, which must not cause any inflammatory, allergic reaction, blood incompatibility, genotoxicity or carcinogenicity to the human body [2,5]. Second, the materials wouldn’t result in any undesirable local or global responses. Third, they should possess high anti-corrosion and anti-abrasive performance, that means the metal ions released by chemical/stress corrosion or abrasion into the human body is low and not harmful for short- or long-term service. Finally, the materials should show good bioactivity to ensure sufficient bonding at the material/bone interface.

**(2) Suitable mechanical properties**

As shown in Table 1, the mechanical parameters of hard tissues are diversified in different positions of the human body. Thus, the materials are expected to possess properties to cover those diversities. First, the strength (compressive, tension, flexure, etc.), fatigue, and wear resistance should be considered. For example, the compressive strength of the bone replacement materials should be higher than 224 MPa [28]. Moreover, the response of the implantable parts to the repeated cyclic loads or strains is mainly determined by the fatigue resistance of the material, which determines the long-term success of the implants that are subject to cyclic loading. In addition, poor wear resistance may also result in the implants loosening and wear debris, which causes the adverse allergic reaction and a reduction in the life-span of the implants. Second, the implantable materials are expected to have relatively low modulus closed to the hard-tissue (0.76–20 GPa) [30,31], because high modulus of implants may cause osteoporosis in the bone around the implants due to “stress shield effect” according to the Wolff’s law, which indicates that the loading on a bone decreases, the bone will adapt and become weaker. Third, the mechanical behavior of the hard-tissue should match the implants to avoid fracture. Some human hard tissues also show the behavior analogy to SE, as shown in Figure 1, and the recoverable strain can reach as high as 2.5% [32]. Thus, the materials for hard-tissue replacement should possess similar or higher recoverable strain (>2.5%) at body temperature [32].

**(3) Microstructural functionality similar to hard-tissue**

The materials are expected to have a similar microstructure with bones or teeth, as shown in Figure 5. They also should possess functionality that is similar to the hard-tissue, such as enough space to allow for cell attachment, spreading, and proliferation, or sufficient nutrient transport towards the cells and removal of waste products. Generally, for bone in-growth, the suitable porosity and pore size ranges from 30% to 90% and 100–500 μm, respectively [36,37]. In addition, homogeneous or gradient porous structure may be required in some body parts, as shown in Figure 5.

## 4. Porous NiTi Shape Memory Alloys

### 4.1. Fabrication and Pore Structure of Porous NiTi SMAs

Although the dense NiTi SMAs are significantly more expensive than the stainless steels and others bio-metals, their unique properties, including SME and SE, non-magnetic and good biocompatibility, make them realize widely biomedical applications, since approved by U.S. FDA in 1989 [38]. However, secondary osteoporosis would cause around the implants due to the modulus mismatching between the dense NiTi SMAs and the hard-tissue. Thus, the porous NiTi SMAs have a great advantage, because their modulus can be adjusted by controlling the pore structure, and they were firstly developed at the end of the 1980s in Russia. We will focus on the biomedical porous SMAs in detail in this section.

In general, porous materials should also meet the following requirements for implanting application. It includes: (1) providing a physically and chemically bioactive surface to promote the cell-material interaction; (2) providing a mechanical function stimulation cell differentiation and biosynthesis, as well as ensuring a temporary or long-term support requirement; and, (3) supporting the development of a three-dimensional tissue by providing a pore structure that is suitable for cell adhesion, proliferation, and differentiation [39]. Moreover, pore structures decide mostly the mechanical properties and biological performances, and thus, play a key role for implantation [40,41]. The pores can be classified into closed and open ones. The closed pores are surrounded by pore walls and isolated from each other, while the open pores are interconnected to each other through various channels. The open porosity ratio is the ratio of open pores to closed ones. Moreover, the porous structure (dense SMAs with porous coating will not be discussed here) can also be divided into the homogeneous porous structure and gradient porous structure. Generally speaking, porous metals with closed pores exhibit mechanical properties that are better than that with open pores at the same porosity [42]. However, the open pores are an often desirable structure for porous scaffolds in bone replacement materials to allow for cell attachment or body fluid exchange [43,44]. Thus, to control porous structure is one basic issue for biomedical porous NiTi SMAs [42,45].

#### 4.1.1. Homogeneous Porous Structure

The porous NiTi SMAs are hard to fabricate by liquid phase processing due to its high melting point (1310 °C). Thus, powder metallurgy (PM) techniques are generally used from either elements powder or pre-alloyed powder [46,47,48,49,50], including conventional sintering (CS) [51,52], hot isostatic pressing (HIP) [53] or CF-HIP [12,54,55], or sintering at a low gas pressure [56], SHS [57,58], as well as spark plasma sintering (SPS) [59] and microwave sintering [60]. Recently, the additive manufacturing (AM), like selective laser melting (SLM) or electron beam melting (EBM), are preferentially used to fabricate porous NiTi SMAs [61,62,63,64]. Because the AM technique has advantages of building porous parts with very complex geometries, it is a very cost-effective, energy efficient, and environmentally friendly manufacturing process [61]. Another special fabricating method is to use a reactive vapor to infiltrate Ni foams [65]. The reader can also refer to the literatures for more description about fabrication method for porous NiTi SMAs [10,48,61]. Table 2 lists the detailed pore characteristics for porous NiTi alloys that are fabricated by various techniques.

**Figure 6:**
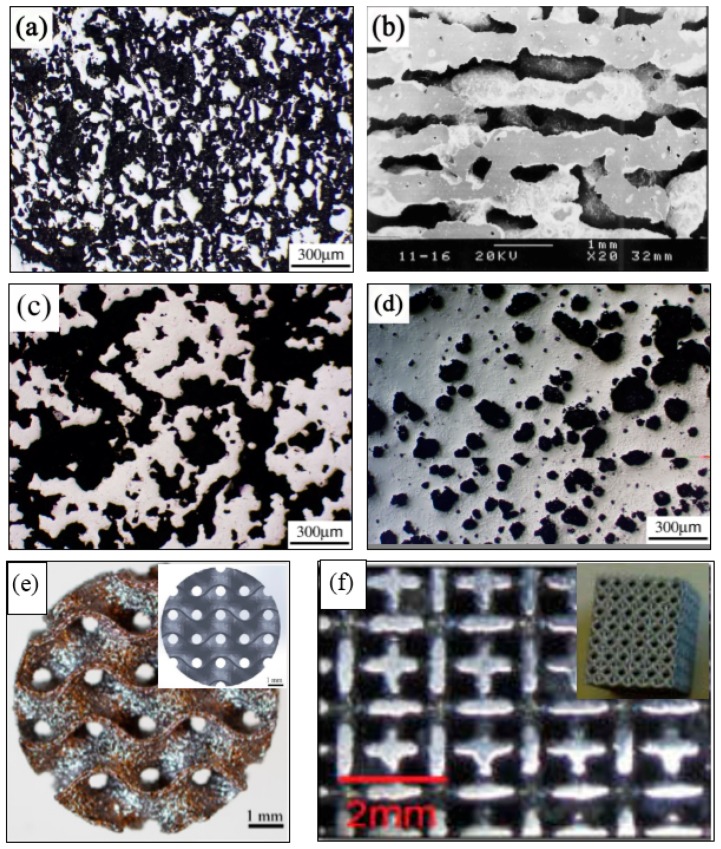
Homogeneous pore structure in porous NiTi SMAs by various methods: (**a**) CS [12]; (**b**) SHS [57]; (**c**) thermal explosion mode in high temperature synthesis (SHS) [12]; (**d**) capsule-free hot isostatic pressing (CF-HIP) [12]; (**e**) SLM (the inset image is the designed part corresponding to the fabricated one) [63]; and, (**f**) SLM (the inset image is a macrograph) [64].

In the CS process, the pressed green samples of Ni-Ti powder mixture is sintered at about 1000 ~ 1050 °C under a vacuum condition or the protection of Ar gas. The inter-diffusion of Ni and Ti atom occurs and forms NiTi intermetallic phase during sintering. However, a small amount of undesirable phase, such as Ni_3_Ti or Ti_2_Ni, are usually formed in the sintered samples due to their lower formation enthalpy, the incomplete reaction between Ni and Ti powders, or eutectoid decomposition of NiTi phase in sintering. These phases are also hard to remove, even by homogenization treatment at high temperature for a long time. In order to reduce those phases, longer sintering time and higher temperature are required, which results in unfavorable severe densification. Thus, the porosity is relatively low (20–50%) and the pore size is small (10–100 μm) for the porous NiTi SMAs fabricated by CS [50,65], as shown in Figure 6a.

For CF-HIP, the green samples are sintered at a pressure much higher than that for CS, usually as high as 150 MPa, which favors the diffusion of Ni and Ti atoms, and the formation of sintering neck, as well as pore structure controlling [54]. Thus, lower sintering temperature and shorter sintering time are usually required in comparison with CS, and wider porosity range (from 10% to 78%) and near-spherical pores (Figure 6d) can be achieved. Furthermore, CF-HIP has the advantage to obtain homogeneous microstructure easily with fewer defects, like cracks, hence higher mechanical properties can be achieved in porous NiTi SMAs [12,66], and we will discuss this aspect in detail later.

Different from CS and CF-HIP, the SHS method forms NiTi phases continuously in entire pressed powder sample by utilizing the exothermic reaction of Ni and Ti from local external energy [58]. Porous NiTi SMAs can be fabricated from the compacted green samples by SHS in a few seconds, and they exhibit relatively high porosity (30–70%), high pore connectivity, and directional pores, which are formed by the gas flowing in Figure 6c. The most disadvantages of SHS are that the processing and the pore characters are hard to be controlled after launching the reaction [67].

In order to control the porosity and the pore characters, the space holders (or pore-forming agent) are added during mixing Ni and Ti powders (or pre-alloy NiTi powders) based on CS, HIP, and SHS, and the space-holders would be eliminated during sintering by degassing or after sintering by selective corrosion [68,69,70]. Various space-holders, such as TiH_2_ [68,71], NH_4_HCO_3_ [72,73,74], carbamide/urea [75,76], NaCl [77], NaF [78,79], Mg [80,81], and even steel wire [82], have been used. Thus, the pore characters with pre-defined size, shape [83], distribution (homogeneous or gradient), and even inner surface can be obtained by controlling particle shape, size, or packing ratio of space holder. It should be mentioned that the porous NiTi SMAs prepared by PM methods with or without a pore-forming agent, usually have high oxygen content with increasing the porosity. It would affect the phase transformation and mechanical properties to some extent.

For AM, the porous NiTi parts can be formed by adding successive layers of the pre-alloyed NiTi powders, rather than removing the materials. Each point or layer is melted or sintered by the laser beam (or electron beam). Any complex or exact porous architectures can be obtained by a three-dimensional computer aided design (3D CAD) method, as shown in Figure 6e, the porous NiTi SMAs is exactly similar to the CAD designed one [63]. Thus, very wide porosity range (0–90%) and any pore shapes (Figure 6f [64]), even complete open porosity can be achieved just by designing [64].

#### 4.1.2. Gradient Porous Structure

**Figure 7:**
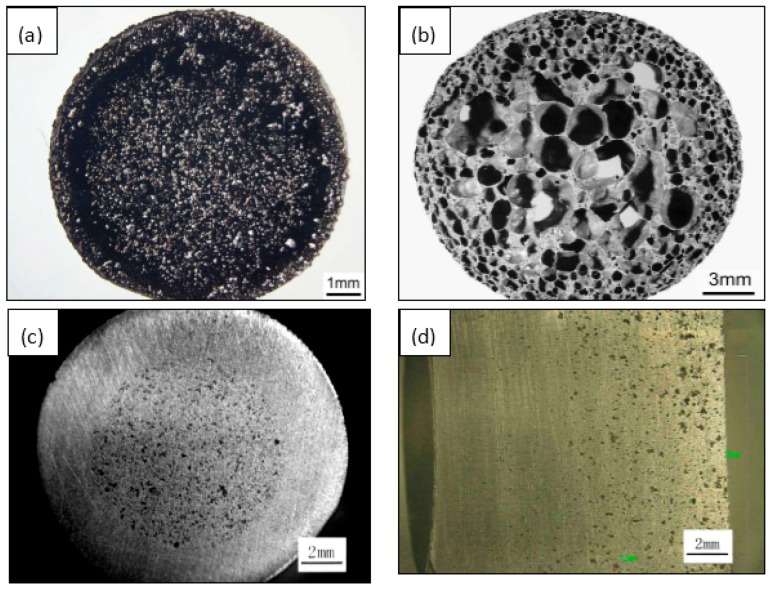
Inhomogeneous pore structure in porous NiTi SMAs: “sandwich-like” structure (**a**), radial gradient structure (**b**) by CF-HIP [84], radial gradient structure (**c**) and axial gradient structure (**d**) by low pressing sintering.

The porous NiTi SMAs fabricated by the above method has a homogeneous structure in general. Anyway, inhomogeneous pore structure can also be obtained by CS with space-holder [74] and CF-HIP. In particular, CF-HIP can conveniently prepare designed porous structure without space-holder [84], such as “sandwich-like” structure and gradient structure. As shown in Figure 7a, three layers structure created in the radial direction, i.e., a porous layer at the outmost, a dense layer in the middle, and another porous layer in the central region, which is similar to that of the femur. With this structure, the outer porous layer is favorable for bone cell adhesion, the intermediate dense layer makes the implants withstand sufficient loads. The sandwich-like pore structure is caused by the combination of high mold pressure and gas pressure during sintering [84]. Figure 7b shows another gradient porous structure by CF-HIP, with porosity as high as 78% and open pore ratio of only about 8%, which is similar to that of one femur bone. This novel pore structure is attributed to the thermal explosion reaction and is formed by the expansion outward of air bubbles in the melting NiTi alloy [84].

**Figure 8:**
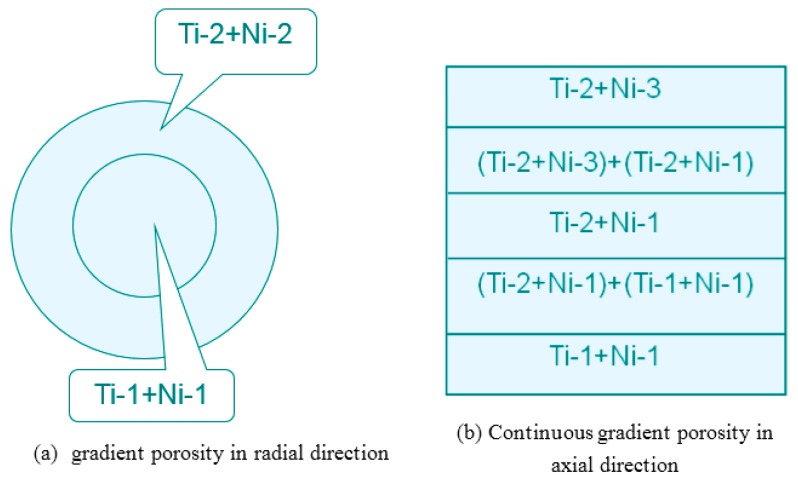
Schematic illustration in the filling of the powder mixture for fabricating two gradient porosity NiTi SMAs (Ti-1 or Ti-2 represents one Ti powder with a certain particle size, the same meaning for the Ni-1 or Ni-2).

The particle size of powder greatly affects porosity and pore size. Porous NiTi SMAs sintered from a designed and mold pressed powder mixture of different particle sizes would exhibit designed porosity and pore size, as schematically shown in Figure 8, Ti-1, Ti-2, Ni-1, or Ni-2 represents Ti and Ni powders of different particle sizes, respectively. Thus, various well controlled gradient porous structures in radial or axial direction can be fabricated in this way. Figure 7c shows an example of gradient structure in radial direction obtained by using the filling way given in Figure 8a, i.e., the outer layer with low porosity (~27%) and small pore size (20–30 μm), while the inner layer with high porosity (~45%) and large pore size (200–300 μm). There is a distinct interface between the inner and outer layer, because of a sharp change in porosity and pore size. By modifying the filling way, as given in Figure 8b, continuous gradient porosity, changing mildly from high (~31%) to low (~21%) along the axial direction, can be obtained and is shown in Figure 7d. Zhang et al. [74] also prepared gradient structure, by CS using space-holder NH_4_HCO_3_ to tune the structure.

Thus, the pore structures resembling the hard-tissue structure, such as a vertebra or femur, can be obtained for biomedical applications.

### 4.2. Microstructure and Properties of Porous NiTi SMAs

**Figure 9:**
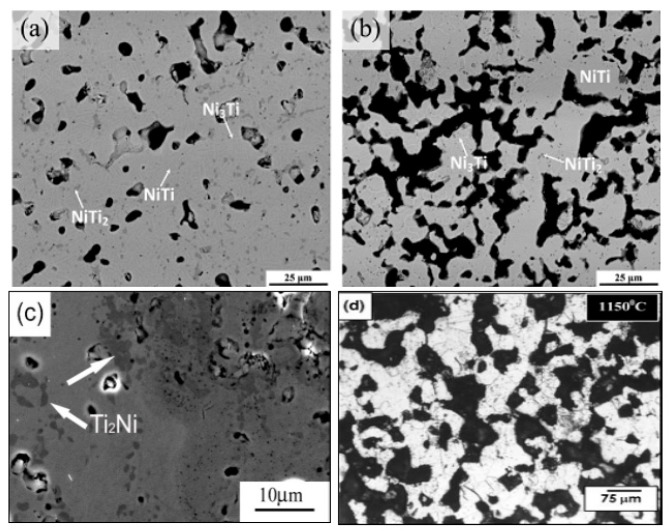
Microstructure of porous NiTi SMAs by: (**a**) conventional sintering (CS) with Ni/Ti powders at 1100 °C for 2 h; (**b**) CS with Ni/TiH_2_ powders at 1100 °C for 2 h [86]; (**c**) CF-HIP, 1050 °C for 3 h [54]; and, (**d**) SHS and post-reaction heat treatment at 1150 °C for 1 h [58].

**Figure 10:**
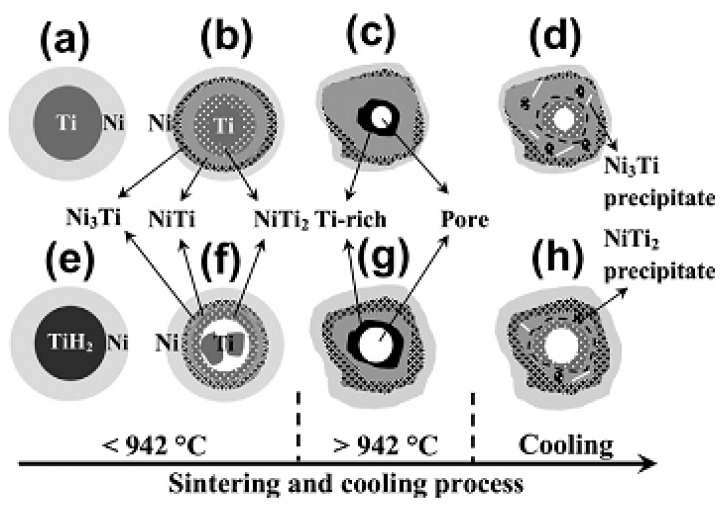
Schematic model of microstructural evolution during reactive sintering and furnace cooling: (**a**–**d**) Ni/Ti; (**e**–**h**) Ni/TiH_2_. Region <942 °C is the solid-state reaction process. Dehydrogenation and separation of newly born Ti powders are shown in (**f**); Region >942 °C is the LPS; Region cooling is final structure below 620 °C after furnace cooling. White lines and black dots indicate the needle-like Ni_3_Ti and spherical NiTi_2_ eutectoid precipitates formed during cooling. [86].

The mechanical and biological performances of porous NiTi SMAs are determined not only by their pore architecture but also by their fine microstructure. For example, the undesirable Ni-rich phases (Ni_3_Ti) or inclusion oxide phases (Ti_4_Ni_2_O_x_) would cause higher Ni ion leaching or deterioration of mechanical and SE [85]. Contrary to dense NiTi SMAs, a small amount undesirable phase, such as Ti_2_Ni or Ni_3_Ti can often be observed even at a high sintering temperature up to 1100 °C in porous NiTi SMAs due to the reasons described in the previous section [85], as shown in Figure 9a,b. By increasing the gas pressure of sintering (i.e., CF-HIP), Ni-rich Ni_3_Ti phases can be eliminated, while Ti_2_Ni phases still present, as shown in Figure 9c. Until very recently, Chen et al. [86] clarified this problem by investigating sintering behaviors from elemental powder mixtures of Ni/Ti and Ni/TiH_2_ while using in-situ neutron diffraction and in-situ SEM. As illustrated in Figure 10, the Ti_2_Ni or Ni_3_Ti phase would form through eutectoid decomposition of B2 phase at 620 °C during furnace cooling when the sintering temperature is over 1000 °C, while they form due to the incomplete reaction between Ni and Ti during sintering below 1000 °C. In order to obtain a single NiTi phase, longer heat treatment time at 1000 °C or higher temperature had to be adopted [58,86], as shown in Figure 9d. Using pre-alloy NiTi powders is also a way to overcome this problem [87], however the cost is inevitably higher.

#### 4.2.1. Effect of Microstructure and Pores on Martensitic Transformation (MT)

As revealed by many studies, the mechanical and phase transformation behavior of the porous SMAs are greatly affected by their microstructure and porosity, in particular at higher porosity [54,66,76,87]. The influences are mainly due to the composition inhomogeneity and the interface effect.

**Figure 11:**
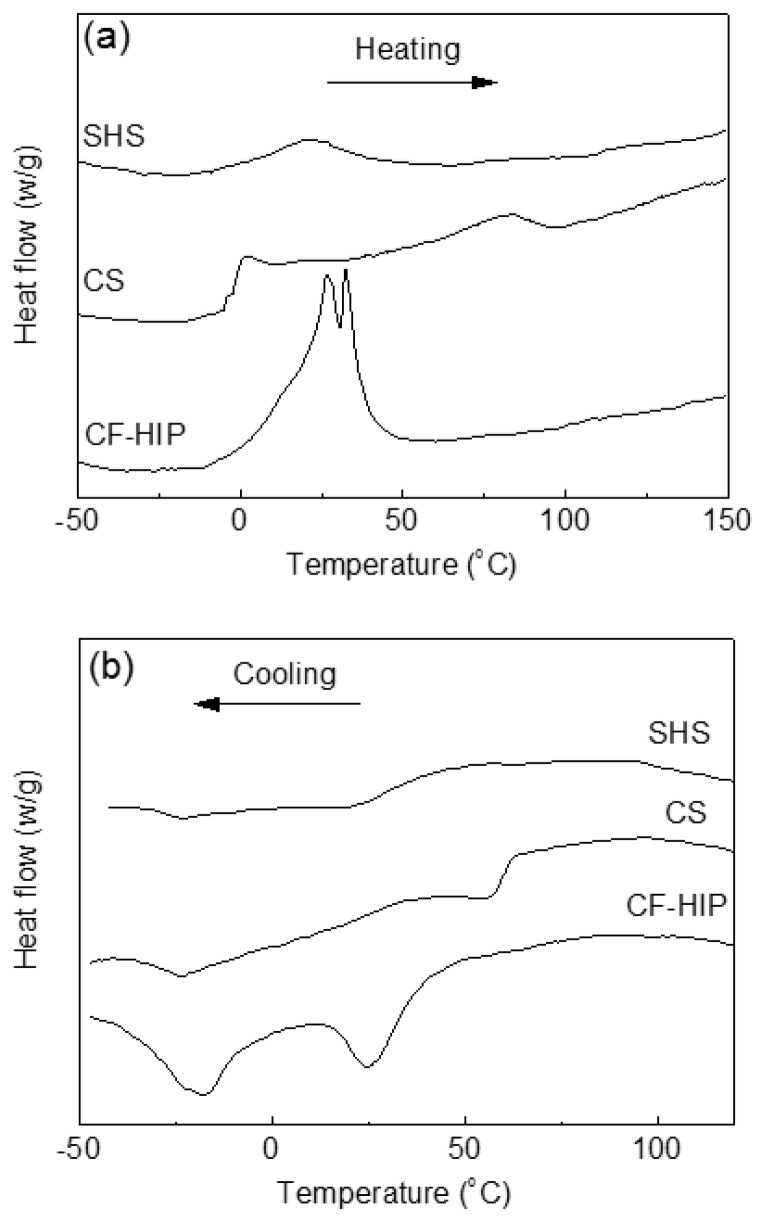
DSC heating (**a**) and cooling (**b**) curves of porous NiTi SMAs fabricated by three methods (CS, SHS, and CF-HIP) [66].

Figure 11 shows the DSC heating and cooling curves of the porous NiTi SMAs that were prepared by CS, SHS, and CF-HIP [66], respectively. It can be seen that the three samples show considerable different transformation behaviors during heating, only one endothermic peak appears in the SHS sample and two distinct endothermic peaks for the CS sample, while two overlapped peaks for the CF-HIP sample. However, it shows two exothermic peaks for all three samples during cooling, despite the difference in the peak position. The above difference in MT behaviors is attributed to the difference in micro-region composition homogeneity of three samples. The porous NiTi SMAs fabricated by SHS show the most homogeneous micro-region composition among the three samples due to the high combustion temperature of ~1300 °C. While the micro-region composition inhomogeneity is the most noticeable for the CS sample. The homogeneity of the composition in the sample by CF-HIP is between that by CS and SHS.

**Figure 12:**
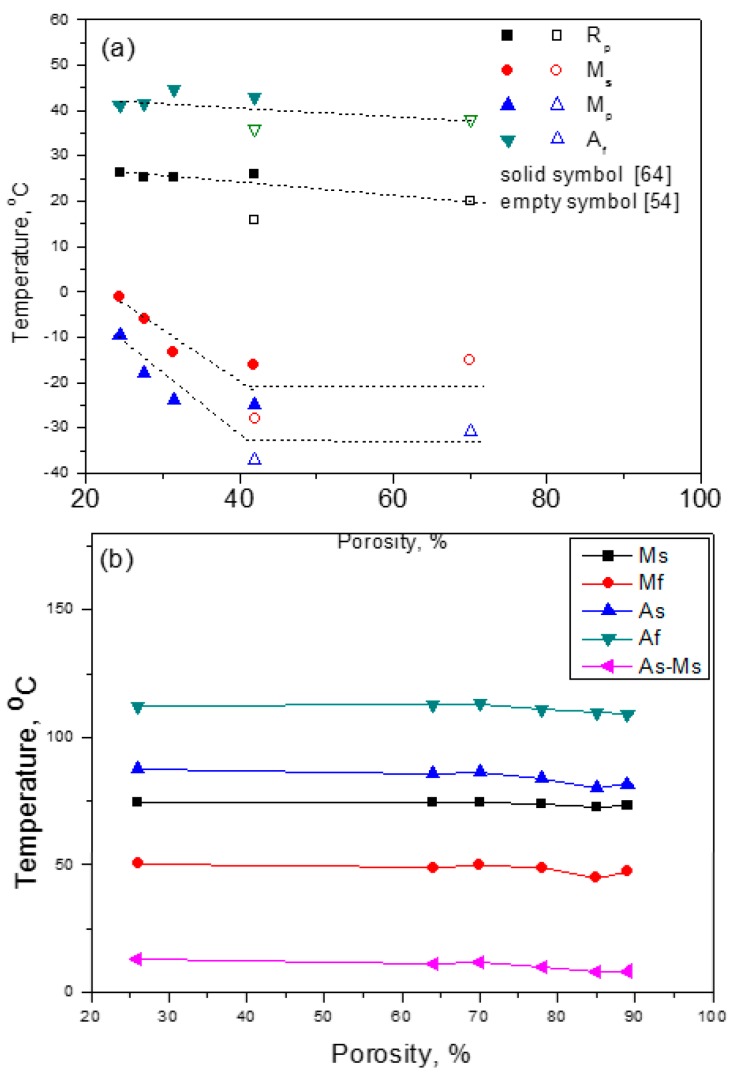
Martensitic transformation temperature dependence of porosity for porous NiTi SMAs with different compositions: (**a**) Ni_50.8_Ti_49.2_ [54,66]; and, (**b**) Ni_49_Ti_51_ [76].

Porous NiTi SMA has a lot of internal pore surfaces, which can greatly affect the MT behaviors because the interfaces, including grain boundaries or surfaces, can promote the nucleation of a new phase. It had been reported that the MT temperatures would change by increasing the porosity for Ni-rich Ni_50.8_Ti_49.2_ SMAs, as shown in Figure 12a [54]. As can be seen, A_f_ and R_p_ of R-phase transformation decrease slightly with increasing porosity, while M_p_ and M_s_ of B19’ MT decrease obviously when the porosity is lower than 40%, and gradually turn steady with further increasing porosity. This is because the quantity of pore walls increases with increasing porosity, and the pore walls promote the nucleation and growth of Ni_4_Ti_3_ particles. Moreover, internal stress would produce around those Ni_4_Ti_3_ precipitations that hinder the formation of B19’ martensite, and thus, reduce the transformation temperature (M_s_ or M_p_) of B19’. In addition, the internal stress around the Ni_4_Ti_3_ precipitations becomes unobvious after growing up to enough size. However, for the Ti-rich porous NiTi alloys, the results are different from that for the Ni-rich ones, as given in Figure 12b, the M_s_ is almost independent of the porosity ranging from 26% to 89% and close to those of dense samples [76]. It should be attributed to no Ni_4_Ti_3_ precipitation formed in the Ti-rich alloys. Thus, the influence of internal stress due to Ni_4_Ti_3_ precipitation seems to be critical.

#### 4.2.2. Effect of Pores on Mechanical Properties and Superelasticity (SE)

It has been reported by many studies [66,86,88,89] that the pores play a very important role in influencing the mechanical and superelastic properties of porous NiTi SMAs, including elastic modulus, critical SIM stress, compression strength, fatigue, and damping behaviors, etc. The reasons are mainly attributed to two factors: (1) the coupling effect between pores and NiTi matrix by stress concentration around pores; and, (2) the reduced valid loading area due to the existing pores. Generally, elastic modulus, compressive stress, and superelastic strain would reduce greatly with increasing porosity.

**Figure 13:**
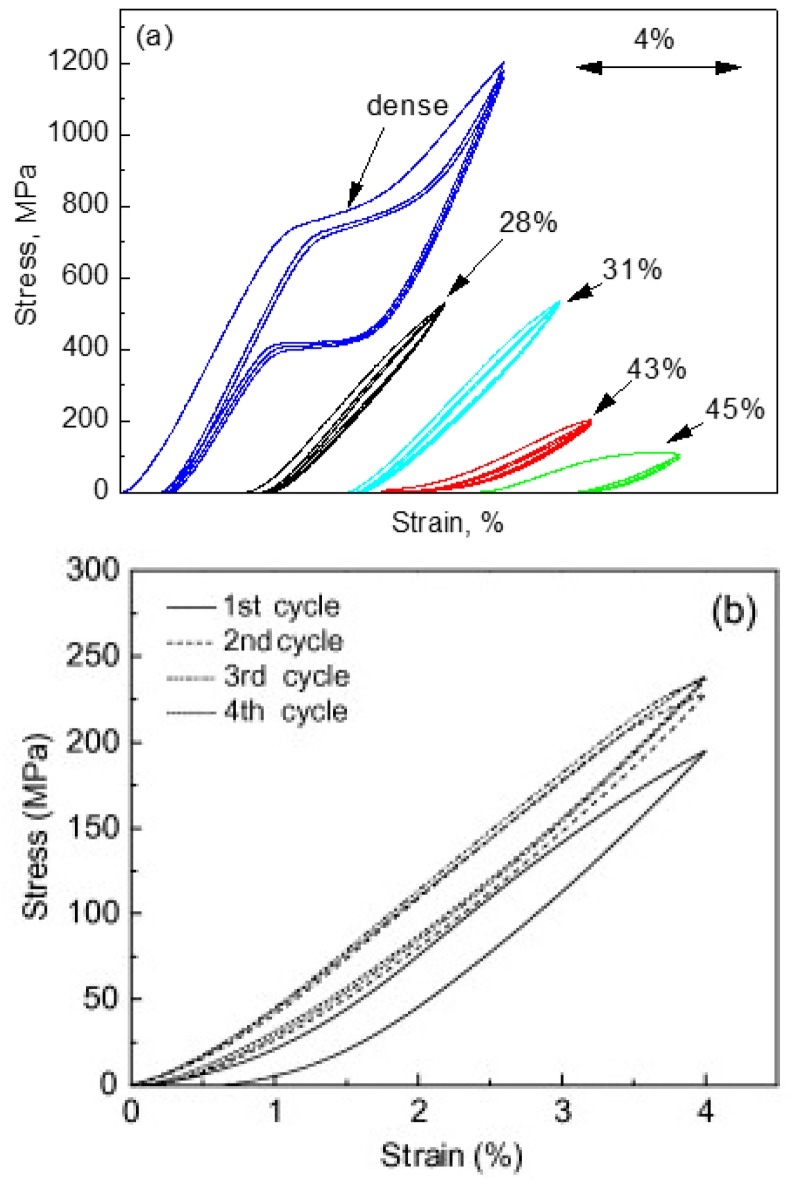
Compressive stress-strain curves: (**a**) homogeneous porous NiTi SMAs by CF-HIP with different porosities at RT; and, (**b**) gradient porosity of 20–61% [74].

**Figure 14:**
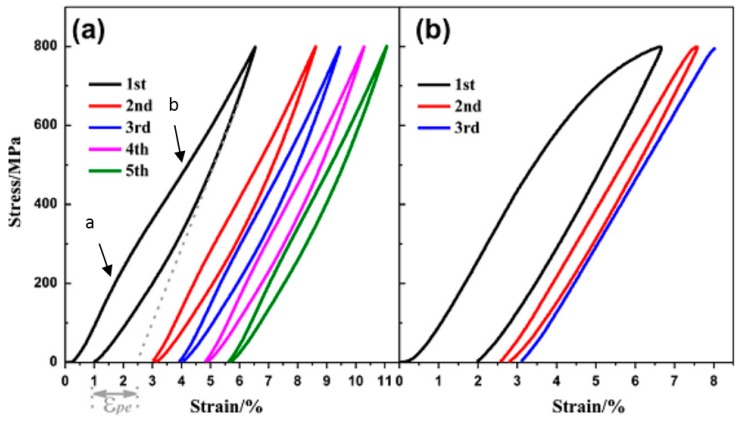
Compressive load–unload recovery cycles under the compressive stress of porous NiTi SMAs by CS with 15% (**a**) and 28% (**b**) porosity [86].

Figure 13 shows the compressive stress-strain curves of CF-HIP porous NiTi SMAs with different porosities in comparison with dense NiTi [88]. The dense NiTi SMAs shows typical compressive stress-strain curves, an obvious SIM plateau of ~700 MPa, and as high as 8% superelastic strain at RT. However, the compressive stress-strain behaviors of porous NiTi SMAs are much different from that of the dense one, i.e., no apparent SIM plateau can be observed in the porous samples with porosity more than 28%, and it shows almost linear elasticity of more than about 4% when the porosity is less than 31%. The result is also confirmed in porous NiTi SMAs by CS, as shown in Figure 14 [86], the sample with 15% porosity show an obvious SIM slope, as indicated by the arrows a and b in Figure 14a, while it is a linear stress-strain behavior for the sample with 28% porosity. The reason can be mainly attributed to the stress concentration around the pores with various sizes as demonstrated by in situ OM observation [89]. The local stress in some micro-regions around the small pores or the tips of pores can possibly reach the critical stress for SIM, even if the applied nominal stress is small. Moreover, the stress concentration factor (the ratio of the maximum stress to the average stress) is related to pore shape [90]. Therefore, SIM occurs continuously in different micro-regions around the pores of different sizes with increasing the nominal stress. Thus, no distinct SIM plateau appears. Recently, Shariat et al. [91] confirmed that SE behavior can be affected by the shape and quantity of pore in NiTi SMAs, and it exhibits the SIM slope over entire forward and reverse MT when 33% of the gauge length is covered by the circular hole.

Furthermore, it is apparent that the slope of the curves (elastic modulus) is gradually reduced with increasing the porosity, the compressive strength and the superelastic strain also decreasing. The porous NiTi SMAs with 45% porosity exhibit only 2% superelastic strain and low compressive strength of 100 MPa, as given in Figure 13a. Similar results had been reported in porous NiTi SMAs that were prepared by other methods [81], for example, porous NiTi SMAs by SHS with about 60% porosity behave like brittle materials and show very small superelasticity. However, porous NiTi SMAs with designed radial gradient pore structure (from 20% porosity in outside to 61% porosity inside) can exhibit superior SE (>4%) and strength (>230 MPa) [74], as shown in Figure 13b. Thus, it is suggested that the concept of gradient porosity will help to develop porous NiTi SMAs with the required pore features. The high porosity and large pore size part would allow and promote tissue cell in-growth, while the denser part would provide crucial SE and sufficient strength.

The porosity selection is highly dependent on different applications, for example, low porosity for load-bearing applications, and a high porosity up to 80–90% only for tissue in-growth. Thus, it is vital to know precisely the mechanical properties and superelastic behaviors (including maximum superelastic strain at RT) for various porosities. For metal foams, it is known that the relationship between the mechanical parameter (P) and porosity (p) can be well predicted by Gibson and Ashby mode, as below, which is based on single phase alloys [48]:

P/P*s* = C(1 − p)^n^ (1)

where P includes the properties of porous metals, such as elastic modulus (E) and strength (σ), the subscript *s* denotes the solid or dense materials, and (1 − p) equals to relative density (ρ/ρ_s_).

However, for porous SMAs, the numerical modeling of mechanical response is still very difficult due to phase transformation during loading and unloading, irregular pore shape and distribution, and complex micro-region internal stress situation around pores. Some researchers attempted to simulate the superelastic behavior of homogeneous or gradient porous NiTi [92] by specific numerical approaches, such as the micro-mechanical averaging technique [93,94,95,96], Eshelby’s effective medium model with Mori–Tanaka mean-field theory [96,97,98], or the Unit Cell Finite Element Method [90,92,99,100,101,102,103]. Most of the simulated relation between elastic modulus (or strength) and porosity agree well with their experimental data in small porosity range. However, almost all of the modeling except few Refs. [96] do not take account for local phase transformation and even plastic deformation around pores. Thus, most of the simulations result obviously do not accord with the observed experimental results of SE mentioned above in Figure 13 and Figure 14.

**Figure 15:**
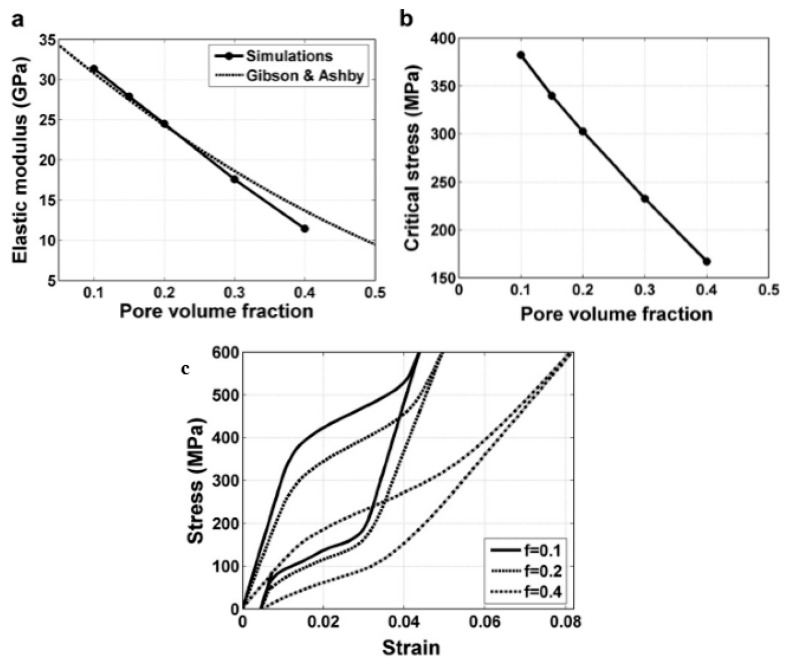
Superelastic behavior of the porous SMAs: (**a**) elastic modulus for the samples with a porosity of 0.1, 0.2, and 0.4; (**b**) macroscopic critical SIM stress for the onset of transformation as a function of porosity; and, (**c**) average stress–strain responses for the samples with porosity. [90].

Panico et al. [90] successfully simulated the complex interaction between porosity, local phase transformation, and macroscale response by adopting the Finite Element Method and taking the development of permanent inelasticity in the matrix into consideration. As can be seen in Figure 15a,b, this model can predict well the trend of elastic modulus and critical SIM stress with porosity, and the results almost coincide with Gibson and Ashby model when the porosity is lower than 30%. Moreover, it can also simulate the difference of superelastic response of porous SMA with various porosities as given in Figure 15c. However, it predicted that the superelastic strain of porous SMAs at high porosity is higher than that at low porosity, which does not agree with the experiment results. Moreover, all of the results cannot interpret the difference of superelastic behaviors between the dense SMA (obvious SIM plateau) and the porous ones (without SIM plateau). Olsen et al. [104] simulated the effect of micro-voids on the superelastic-plastic behavior of NiTi SMA. They found that the average stress level lowers, and both phase transformation and plastic yielding occur at considerably lower stress by introducing micro-voids in a homogeneous material. In addition, it is found that the superelastic stress–strain hysteresis becomes narrower with increasing porosity, i.e., the amount of energy dissipated during a stress–strain cycle is reduced. Hence, further studies need to be carried on about the coupling effect of pores on the performance of porous NiTi SMAs.

**Figure 16:**
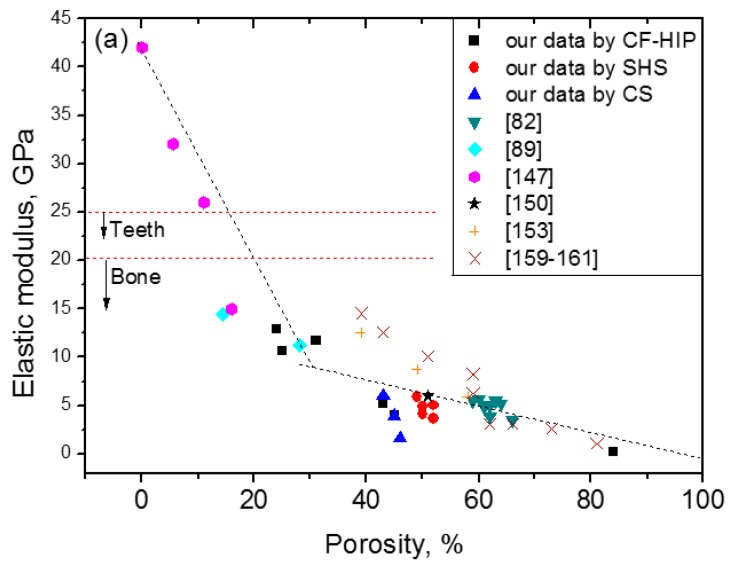

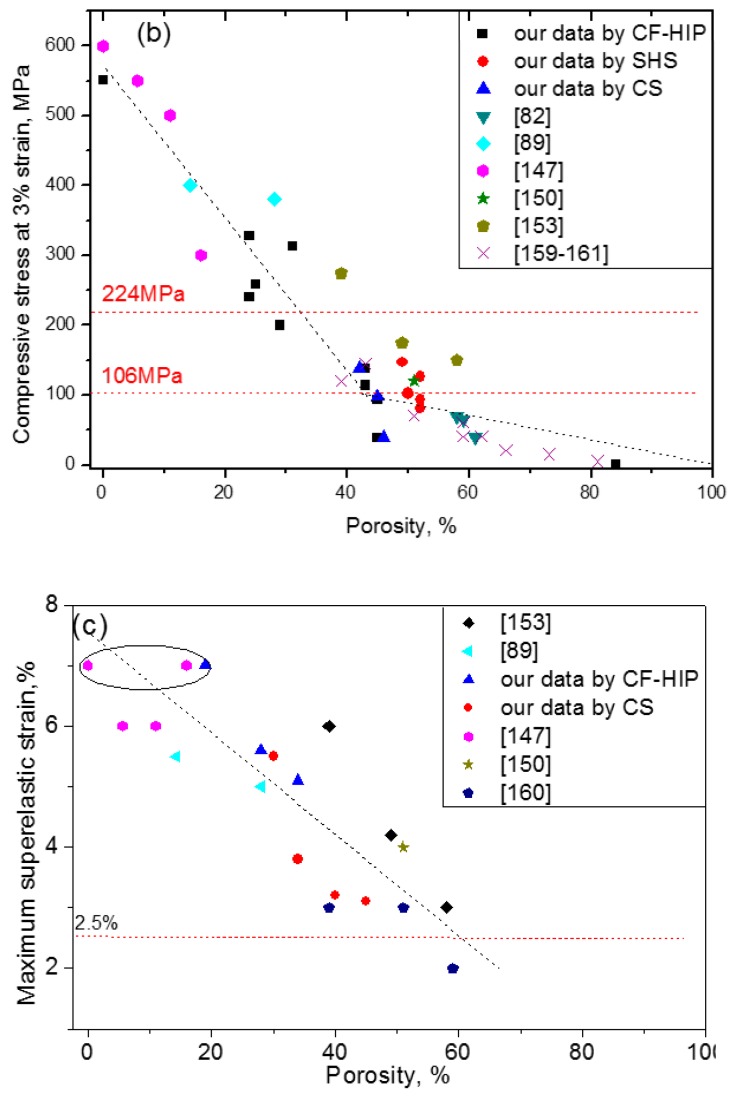
The relationship between elastic modulus (**a**), compressive stress at 3% strain (**b**), maximum superelastic strain (**c**) and porosity for porous NiTi (Ti_49.2_Ni_50.8_) SMAs prepared by various methods (data from refs. [160,161,162] are Ti_49.4_Ni_50.6_).

Here, the relationship between the porosity and elastic modulus, compressive stress at 3% strain (slightly higher than 2–2.5%, the maximum recoverable strain of cortical bone), and the maximum superelastic strain were summarized and plotted in Figure 16 based on the experimental results for porous NiTi (Ni-rich) SMAs. This plot can be used as a guide for selecting porous NiTi SMAs for different applications. It indicates that the dependence of elastic modulus on porosity cannot be expressed by the single linear model (or Gibson and Ashby model), but it can be divided into two stages, as given in Figure 16a. The E (elastic modulus) rapidly decreases with increasing porosity in the range smaller than ~30%, while the tendency turns slowing in the porosity range higher than 30%. It can be seen that the elastic modulus of porous NiTi SMAs can be adjusted to match that of cortical bone (6–20 GPa), or cancellous bone (<4 GPa) when the porosity is higher than 20%. The same trend can be found for the compressive stress, as shown in Figure 16b, and the critical point is also ~35% porosity. Although the physical meaning of this critical point is still unclear, it may relate to the change of the pore structure from closed pores dominating to open pores dominating, and thus more martensite (lower modulus) cannot revert back to parent phase (high modulus) due to severe stress concentration around open pores. It is no doubt that porous NiTi SMAs with a porosity lower than 40% can fulfill the requirement for the maximum compressive stress of 224 MPa for cortical bone [28].

However, the relationship between maximum superelastic strain and porosity is different from that for elastic modulus and compressive stress. For all the reported results (the porosity changes from 0% to 60%), the maximum superelastic strain almost decreased linearly with increasing porosity and would decrease to below 2.5% when porosity is higher than 60%. It should be noted that porous SMAs with porosity smaller than 20% exhibit almost the same superelastic strain as that of the dense ones.

Thus, after evaluating all those three factors, it can be concluded that porous NiTi SMAs with a porosity of 30–40% can be considered as replacement materials for cortical bones, while that with 40–90% porosity can be used in cancellous bones. Furthermore, porous NiTi SMAs with gradient porous structure (or sandwich porous structure) can further satisfy the requirements of various hard-tissues.

**Figure 17:**
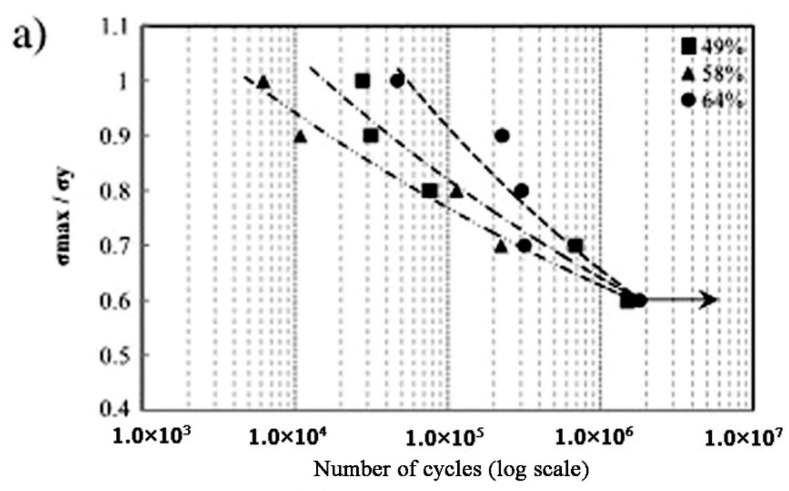

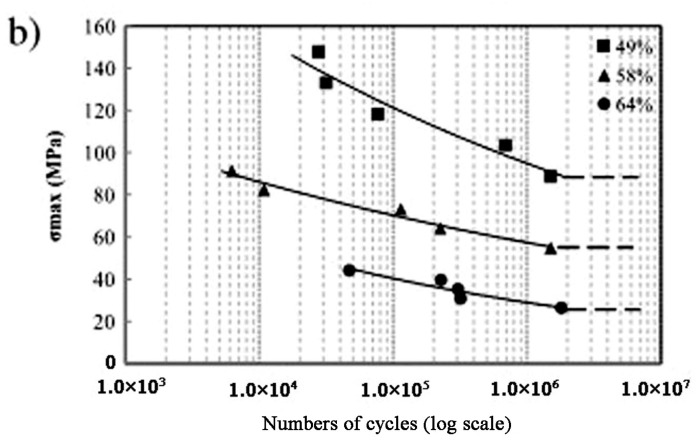
The S-N curves for porous NiTi SMAs with different porosities in terms of (**a**) the yield value and (**b**) the maximum applied stress [105].

Besides the above three important factors, the fatigue is one vital issue to be considered in biomedical applications [105,106]. Kohl et al. [107] reported that the maximum stress and SE of porous NiTi SMAs with a porosity of 51% decrease with increasing the number of cycles, the SE strain is smaller than 1% after 100000 cycles. The fatigue deterioration is due to the microcracking from the sharp notches of irregular pores and the brittle Ti_4_Ni_2_O_x_ phase. By forming near spherical pores in porous NiTi SMAs by CF-HIP [108], the fatigue is greatly improved with very good deformation recovery ability in term of a linear SE as high as 4%. Moreover, when the high cycling strain is applied, the degradation of superelasticity effect only occurs in the first cycle, the good linear SE is maintained thereafter. Nakas found that the endurance limit or the critical stress for failure decreases with increasing the porosity [105,106], and it is worth to mention that these values for all the porosity (49–64%) are above the critical stress level that an implant is usually subjected to, as shown in Figure 17 [105]. Obviously, to remove the undesirable phases (Ti_2_Ni or oxide) and to obtain near spherical pores should benefit fatigue performance of porous NiTi SMAs.

The abraded debris might cause osteocytic osteolysis on the interface between implants and bone tissue, and thus inducing the subsequent mobilization of implants gradually, and finally resulting in the failure of bone implantation. For example, Rhalmi et al. [109] simulated the event of fatigue debris released from porous NiTi SMAs, and evaluated the toxicity of alloyed NiTi particles indirectly contact with surrounding tissue, particularly spinal cord dura matter. The implanted NiTi particles caused inflammation (acute at first and reducing to mild chronic over time) only at the adjacent epidural space, while the abnormal response from the dura matter was maintained over one year of implantation.

**Figure 18:**
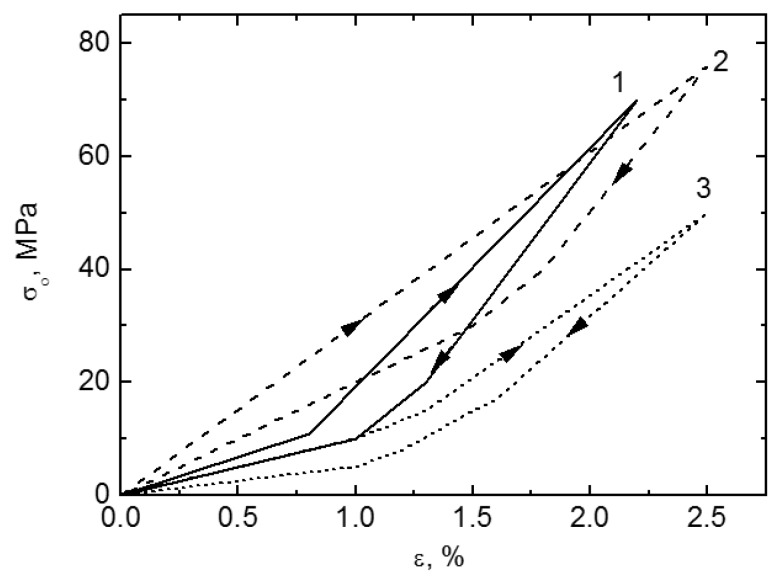
Loading-unloading curves for a sintered porous NiTi implant (Φ4 × 4 mm^3^ cylinders) deformed in compression mode before implantation (curve 2), after one month (curve 3) and after three months (curve 1) of implantation in rabbits [110].

Besides the above mentioned in vitro properties, it is more important to evaluate in vivo mechanical and biomechanical performances of porous NiTi SMAs. Although other porous implants, such as porous stainless steel, porous Ti, and porous HA, can relieve stress shielding effect by matching elastic modulus with hard-tissue, the SE of porous NiTi SMAs has significant advantage over others porous biomaterials imitating the mechanical behaviors of hard-tissue, such as bones or tendons, which has been shown in Figure 1. By investigating the porous NiTi composited with bone implanted in rabbits for one and three months, as shown in Figure 18, Itin et al. found that the mechanical performance of the composite of porous NiTi and bone is even superior to either porous NiTi or bone alone [110]. The SE of porous NiTi SMAs can be maintained even after bone ingrowth satisfying the hard-tissue requirement for biomechanical compatibility. Porous NiTi SMAs with 40–50% porosity can exhibit more than 3% recoverable strain, which is greater than bone’s own recoverable strain of about 2.5%. Thus, the implant is more likely to keep integrated with the bone that is subjected to the physiological peak stress when a person is climbing stairs (870% body mass), which may deform the bone beyond the elastic deformation limits of implant materials in current use.

#### 4.2.3. Effect of Pores on Biomedical Properties

**Figure 19:**
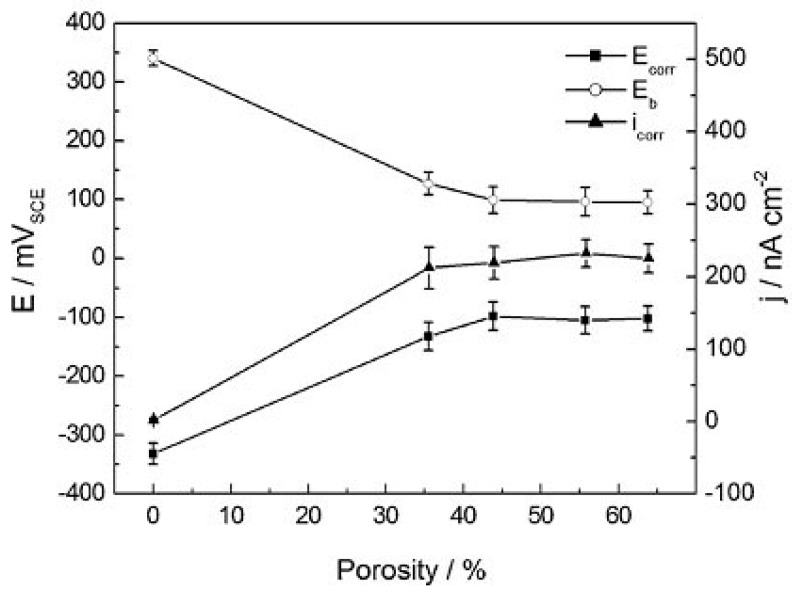
Extracted electrochemical parameters from polarization curves in a 0.9% NaCl solution for 24 h with porosity in porous NiTi SMAs [110].

**Figure 20:**
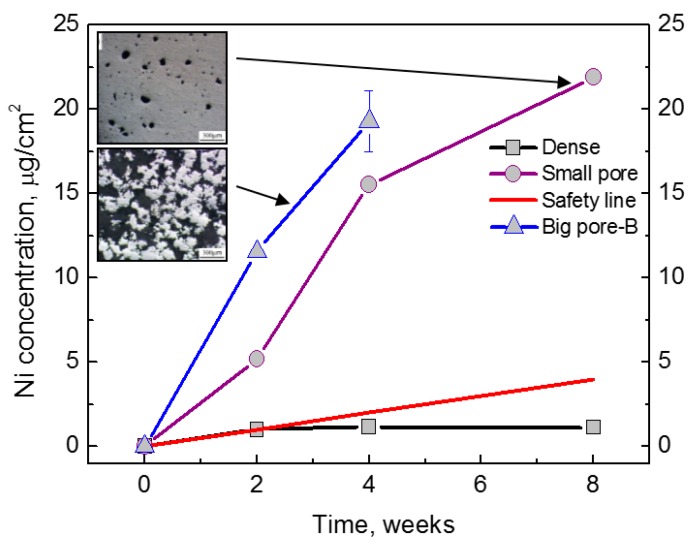
Ni ion releasing content of porous NiTi SMAs by CF-HIP with different pore sizes dependence of immersion duration (the safety line represents for the acceptable Ni ion content to the human body 0.5 μm/cm^2^/week [111]).

In comparison with other bone graft substitute materials, porous NiTi SMAs possess satisfactory biomechanical properties. Thus, their biological properties, including corrosion resistance and biocompatibility, become a critical issue for hard-tissue biomedical applications. In fact, the corrosion resistance, cell attachment or in-growth would be greatly affected by the pore structural characteristics, such as porosity (or pore volume), pore size, and pore shape. Many investigations [39] had proved that high specific surface area of porous SMAs have a great effect on the corrosion properties, Ni ion releasing, bone osteointegration, etc., but the results are controversial, which may be attributed to different surface status. Itin et al. [110] demonstrated that the corrosion rate of porous NiTi SMA by SHS is closed to that of dense one, and are much lower than that by CS because the SHS alloy has low surface area due to larger pore size. However, the corrosion rate almost does not further increased in porosity range higher than 35%, as shown in Figure 19. It has been also found that Ni ion releasing of porous NiTi SMAs is ten times higher than the dense one in simulated body fluid (SBF) [111], as shown in Figure 20. Furthermore, the released Ni ion content is affected by pore character and is much higher than the safety line (0.5 μg/cm^2^/week) [112]. However, it has also been reported that porous NiTi SMAs show good resistance to local and general corrosion by potentiodynamic polarization evaluation and low Ni leaching [113].

In general, as proved by many studies [62,114,115,116], porous NiTi SMAs exhibit good cell adhesion and biocompatibility, which also depends on the structure of surfaces. For example, Assad et al. [117,118] systematically studied the biocompatibility of porous NiTi SMAs by in vitro and in vivo testing, and concluded that the short-term biocompatibility of porous NiTi is comparable to that dense NiTi, moreover it has no potential to produce irritation, systemic toxicity reactions, or sensitization in animal models by in vivo standard allergy potential evaluation.

In addition, porous NiTi SMAs also exhibit good tissue biocompatibility in vivo by some investigation [40,119,120]. No apparent adverse reactions were observed around the implanting areas in the proximal tibia of the rabbit after six weeks, and the in-growth bone has similar properties to the surrounding bone [121]. Moreover, it shows excellent osseointegration and bony contact in rabbits and rats without signs of loosening. For example, it has been found that CF-HIP prepared porous NiTi exhibit good cytocompatibility and good superelastic biomechanical properties in comparison with dense NiTi and porous pure Ti, and thus are considered to be suitable for load-bearing hard-tissue replacement materials [122].

It was also reported that the porosity and pore size of biomaterial scaffolds play a critical role in bone formation in vitro and in vivo [123]. Large pores and high porosity can promote bony contact and tissue in-growth, and improve the fixation and remodeling between the implantation and the human tissue [119], and therefore, can improve the biocompatibility of porous NiTi SMAs. By in vivo evaluation, for example, Kujala et al. [40] found that the SHS porous NiTi SMAs with high porosity show best bone contact, while the sample with the lowest porosity show a lower incidence of fibrosis within the porous NiTi, although bone contact is significantly inferior. Moreover, Ayers et al. [119] also found that porous NiTi SMAs with higher pore volume (or specific surface area) exhibit a higher volume of ingrown bone and external bony apposition. However, Ayers et al. [119] also addressed that there is no apparent correlation between pore size (for 100–400 μm) and bone in-growth near the interface during the cartilaginous period of bone growth for porous NiTi SMAs, because the thickness of implants is the same order as the pore size.

In summary, porous NiTi SMAs has great potential as biologically safe materials for hard-tissue replacements.

### 4.3. Surface Modification of Porous NiTi SMAs

Ni is in a tightly bound with Ti in NiTi alloys, Ni leaching is tiny and safe for dense NiTi SMAs with proper treatment after implantation for long-term, which is admitted by FDA. For example, in vivo Ni concentrations in the tissue around the NiTi implants are reported to be approximate 0.2 µg·g^–1^ in animal studies [40], and concentrations of Ni ions had been found in vitro in a range of 10 µg·g^–1^ to inhibit cell growth [121,122,123]. However, it is still a concerning issue that excessive Ni ion would release inevitably from the matrix materials due to corrosion or abrasion in the biological environment and induce adverse influences, such as allergic response, cellular hypersensitivity, cytotoxicity, and genotoxicity, and even endanger life [120]. With respect to porous NiTi, Ni ion releasing is of particular concern due to very high exposed surfaces, although porous NiTi SMAs shows great advantages in biomedical applications [116]. It is known from Figure 20 that the Ni ion releasing from porous NiTi is one or two orders of magnitude higher than that from dense NiTi [111,124]. Furthermore, the Ni leaching would be greatly enhanced because of the unavoidable presence of Ni_3_Ti (even pure Ni) in the sintered porous NiTi SMAs [86]. Thus, it is a vital issue to reduce the Ni ion releasing. Surface modification is an ideal method to reduce Ni leaching while keeping its other excellent performances, and a lot of investigations [125,126,127,128,129,130,131,132,133,134,135,136,137,138,139,140,141,142,143,144,145,146,147,148,149,150,151,152,153,154,155,156,157] have been made in recent ten years.

Surface modification of dense NiTi SMAs generally aims at forming an enhanced corrosive protective layer to prevent Ni releasing. Moreover, some special consideration, such as a bioactive layer allows cell attachment combining with abrasive resistance property, would also be taken into account. Thus, various surface treatments, including surface finishing [125,126,127,128,129], passivation [130,131,132,133,134,135,136,137,138,139,140], coating [145,146,147,148,149,150,151,152,153,154,155,156,157], and sterilization [158], have been developed, and their effect on corrosion resistance and Ni leaching level in vitro and in vivo, as well as biological performances, have been investigated. Those protective films on the surfaces of NiTi SMAs include mainly bio-inert layers (such as Ti oxides [130], TiN [148], TiC [149], etc.) and bioactive layers (for example HA [152] or others [154]).

Obviously, surface modification is even more necessary for porous NiTi SMAs because of its higher corrosion rate and Ni leaching level, as mentioned before [110,112]. However, some surface modification methods applied in dense NiTi SMAs are invalid for porous ones because of the complex pore architecture (interconnect pore channel is usually smaller than 50 μm), and obstruction for inner surfaces of closed pores. For example, chemical vapor deposition (CVD), physical vapor deposition (PVD), or plasma immersion ion implantation (PIII) is applicable to exposed surfaces but not to inner pores. Thus, thermal oxidation [114], wet chemical oxidation [159,160], or gas nitriding [65] were attempted for the surface modification of porous NiTi SMAs.

Thermal oxidation in air is a valid surface treatment for the complex interconnected pore surfaces. Wu et al. [114] found that an optimal parameter is annealing at 450 °C for 1 h, and the treated porous NiTi SMA exhibits a corrosion rate that is two orders of magnitude lower than the untreated one. However, it would cause much higher Ni leaching level (a factor of 12–25) than the untreated NiTi due to the formation of thicker Ni-rich layers under the thin TiO_2_ layer. Significant results were also achieved by using PIII method. Ho et al. [161] reported that Ni leaching of the porous NiTi reduced to one-third of the original one after treated by Oxygen PIII. Moreover, a high corrosion resistance was shown and even comparable to untreated dense NiTi. In addition, the material behaves the same SE as the untreated one [162].

**Figure 21:**
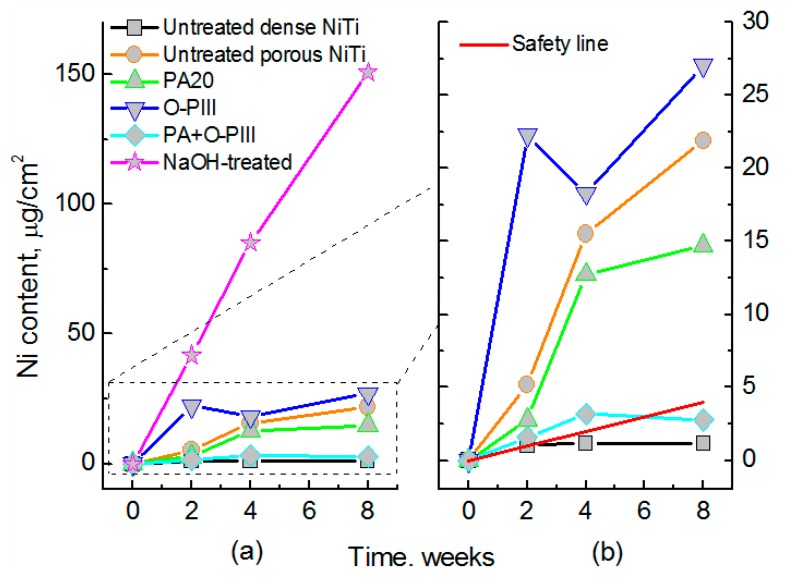
Ni leaching level of porous NiTi treated by different wet chemical treatments (**a**), in comparison with untreated NiTi and safety Ni level to human body; (**b**) is an enlarged area from (**a**). (PA: passivation in HNO_3_; O-PIII: Oxygen PIII; NaOH-treated: treated in NaOH solution) [111].

The wet chemical oxidation is also effective in enhancing corrosion resistance, as well as in depressing Ni leaching [159,163,164]. For example, Jiang et al. [163] reported the formation of crystalline HA layers on porous NiTi (after five days, which is faster than natural HA formation) by wet passivation and subsequent immersion in SBF. The resulting HA layer uniformly covered the porous NiTi, both on the surfaces and within the pores, and Ni release was even lower than that of untreated dense NiTi (up to 50 days). Gu et al. [159] reported the faster formation of apatite layer on the surface of porous NiTi SMAs with nearly circular pore shapes. Wu et al. [160] made a large-scale direct growth of nanostructured bioactive titanates in porous NiTi SMAs via a facile low temperature hydrothermal treatment in NaOH solution, and the modified surface shows superhydrophilicity and favors the deposition of hydroxyapatite and accelerates cell attachment and proliferation. Gotman et al. [65] modified porous NiTi (70% open porosity) by nitriding, and its Ni leaching is an order of magnitude lower than that without surface treatment. However, most studies didn’t compare the Ni leaching level with the acceptable one for the human body, although most results were concluded to be positive. Thus, Yuan et al. [111] compared different wet chemical oxidations, and found that all of those porous NiTi samples treated by single wet surface treatment exhibit Ni leaching level higher than the safety line, except the one treated by the combining HNO_3_ passivation and oxygen PIII, which can be depressed under the safety line beyond six weeks, as shown in Figure 21.

**Figure 22:**
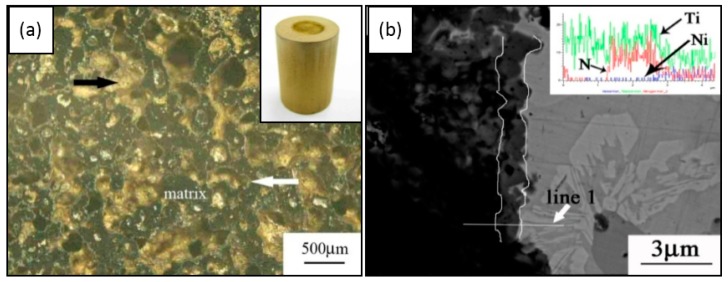
Optical image (**a**) and SEM image (**b**) of porous NiTi SMAs fabricated by sintering and in-situ nitriding (inset image in (**a**) is a macrographic image) [165].

**Figure 23:**
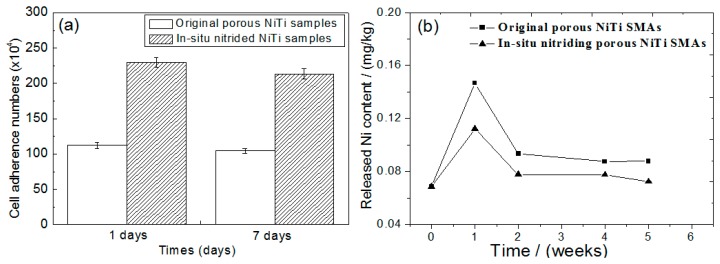
In vitro cell attachment (**a**) and in vivo Ni leaching (**b**) porous NiTi SMAs treated by in situ nitriding comparing with untreated porous NiTi [165].

Obviously, all those above mentioned surface modifications can only form a protective film on the outer surface and the inner surfaces of open pores, but they are invalid for the inner surfaces of closed pores. Thus, the concern would be raised for the increasing of Ni ion leaching if the closed pores crack and more fresh untreated surfaces are exposed to body fluid and accelerate corrosion during cyclic loading. Thus, some attempts have been made to solve this problem. Berthville [52] proposed to fabricate porous NiTi by SHS under an atmosphere of Ca vapor, the pore surfaces would be coated with residual calcium oxide. It may promote bone in-growth if calcium oxide changes to Ca(OH)_2_, but further results have not been reported. Recently, Li et al. [165] developed an in-situ nitriding method through the decomposition of ammonia during sintering. A TiN protective layer of 1μm thick can be formed on all surfaces, including closed pores, as shown in Figure 22. The porous NiTi treated by this way, as the in vitro and in vivo results shown in Figure 23, exhibit higher cell attachment number and lower Ni releasing level than the untreated one.

Based on the above discussion, free of undesirable Ni-rich phases and the homogeneous protective layer is a pursue goal for surface modification of porous NiTi SMAs. However, the assessment of the durability and stability is still lacking for the surface protective layers under cyclic loading or body fluid environment, in particular, for their long-term performance. In addition, the effect of surface treatments on the overall SME and SE behavior of porous NiTi SMAs should be also emphasized.

### 4.4. Application of Porous NiTi SMAs

**Figure 24:**
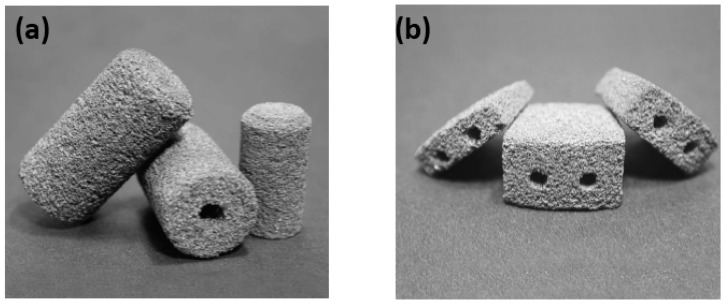

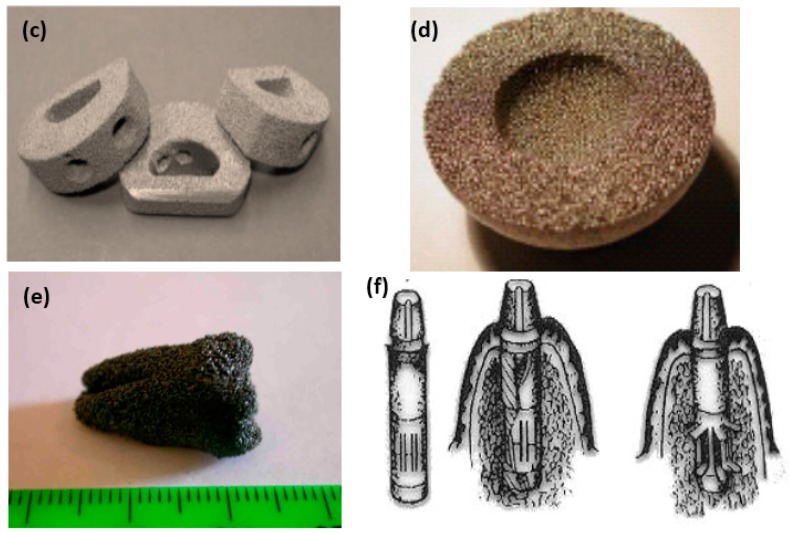
Biomedical products made by porous NiTi SMAs: (**a**) Cervical spine implantation [169]; (**b**) lumbar spine implantation [169]; (**c**) intervertebral fusion device [169]; (**d**) acetabular cup [167]; (**e**) tooth [170]; and, (**f**) gum tissue replacement [167].

In addition to the unique SME, SE, biocompatibility, and biomechanical compatibility, porous NiTi SMAs also possess additional benefits common to other porous metallic materials, such as low density, high specific surface area and high permeability. Furthermore, porous NiTi SMAs have additional advantages of excellent compatibility for magnetic resonance imaging (MRI) and computer tomography (CT) scanning [166]. Thus, it has already been proposed to using them in many aspects, including biomedical applications [167], energy absorption [168], light-weight actuator [102], etc. Till now, various biomedical applications in bones or teeth, such as maxillofacial repairing, bone fixation plates or screws, teeth root replacements [168], acetabular cup, femoral stem replacements, and cervical and lumbar vertebral implantation [167] are still the main targets for porous NiTi SMAs. For example, some commercial porous NiTi products had fabricated by SHS in Canada (Biorthex Inc., Boucherville, QC) for spine implantations and replacements, as shown in Figure 24a–c, and they already have more than hundreds of clinic application examples since 1984 [169]. One of the advantage of porous NiTi SMAs as compared to other porous metals for implanting (e.g., pure Ti and stainless steel) is the excellent biomechanical compatibility (i.e., SE) of NiTi, as shown in Figure 1. In addition, the acetabular cups also can be manufactured while using porous NiTi SMAs, they possess the similar porous structure, elastic modulus and mechanical behavior with the surrounding bones. Thus, the patients after implantation would recover at a shorter time and without secondary osteoporosis due to SE. Shishkovsky et al. attempted to fabricate tooth while using porous NiTi SMAs by selective laser sintering (SLS), as shown in Figure 24e [170]. Moreover, one of the advantage of porous NiTi SMAs as compared to other porous materials is the firm fixation of implanting teeth by using SME. For example, the gum tissue part can be deformed into a cylindrical shape before surgery, as shown in Figure 24f, it can be smoothly implanted into the gum of the patient, and then it can be firmly fixed at body temperature due to its unique SME.

## 5. Porous Ni-Free Shape Memory Alloys

No matter dense or porous NiTi SMAs are considered, the Ni ion issue seems always a vital concern for the doctors and patients, although Ni ion releasing had been proved to be safe in the human body within relatively short-term (e.g., <10 years) after various surface modifications. In addition, there are some uncertainties for surface protection, in particular for porous NiTi SMAs due to complex pore architecture and the various stresses and physiological conditions. Thus, a great deal efforts are being made to develop Ni-free SMAs to ultimately solve the Ni releasing problem in recent years.

### 5.1. Dense Ni-Free SMAs

#### 5.1.1. Development History and Alloy Systems

As well known, pure Ti and Ti alloy have been used as hard-tissue replacement materials because of their excellent biocompatibility and corrosion resistance, as well as low density, in comparison with other biometals, such as Co-Cr and stainless steels [5,16]. Ti alloys are generally categorized into three different groups of α, β, and (α + β)-type alloys. Ni-free SMAs only exist in β type Ti alloys because the β phase (BCC crystalline structure) transforms to either metastable α′ (hexagonal structure) or α″ martensite (orthorhombic structure) by quenching from high temperature, and the β to α″ transformation is a reversible MT that leads to the SME [171]. The transformation temperature can be tuned by adjusting alloy composition [172]. However, these β type Ti-based SMAs haven’t attracted much attention until recent ten years because of wide applications of NiTi SMAs in numerous biomedical areas.

Until now, SME or SE have been reported in several Ni-free β type Ti-based binary alloy systems, including Ti-Nb [173,174,175,176], Ti-Mo [177,178], Ti-V [179], and Ti-Fe [180,181]. However, these binary Ti-based SMAs exhibit poor SME stability and small recoverable strain (generally <3%) when comparing with NiTi SMAs of 8% recoverable strain. Thus, in order to improve their SME or SE, alloying elements are added into those binary alloy systems. Many ternary even quaternary Ni-free SMAs have been developed, including Ti-Nb-based (such as Ti-Nb-Zr [176], Ti-Nb-Ag [173], Ti-Nb-Al [174], Ti-Nb-Ta [182], Ti-Nb-O [183], Ti-Nb-Fe [184], etc.), Ti-Mo-based (such as Ti-Mo-Ga [177], Ti-Mo-Sn [185], etc.), Ti-V-based (such as Ti-V-Fe-Al [186], Ti-V-Cu-Cu-Co-Al [187], etc.), Ti-Fe-based (Ti-Fe-Nb [181], Ti-Fe-Ta [180], etc.), and Ti-Zr-Nb-based [188].

Among those Ni-free Ti-based SMAs, Ti-Mo-based alloys are susceptible to ω phase embrittlement, and Ti-V-based alloys are not suitable for biomaterials due to the cytotoxicity of V [189]. However, Ti-Nb-based alloys [173,174,175,176,190,191], including Ti-Nb-Zr and Ti-Nb-Ta alloys, etc., attract more attention, because Nb, Zr, and Ta can lower modulus and increase the strength of β type Ti alloys, in addition to their non-toxicity. Thus, this review focuses on the effect of alloying elements and heat treatment on microstructure, SME, SE, mechanical, and biological properties of Ti-Nb-based SMAs.

#### 5.1.2. MT, Microstructure and Shape Memory Effect (SME)

The fabricating methods and heat treatment process of Ni-free Ti-based SMAs are actually similar to that for β type Ti alloys. Generally, the fabrication of dense Ni-free Ti-based SMAs with fine grain size or desirable texture, consists of melting, cold or hot rolling of the ingot, and the following annealing or aging heat treatment, and will not be further described in the following text, except that the special process is involved.

**Figure 25:**
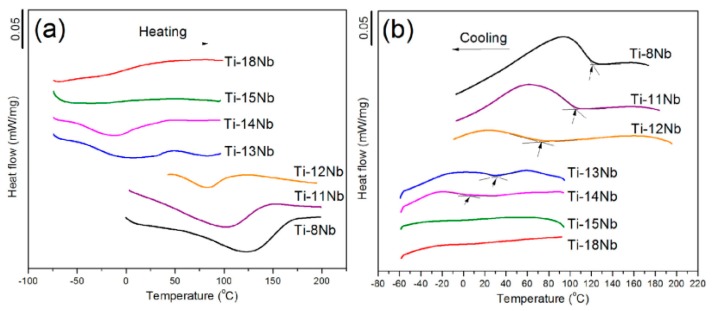
DSC curves of the sintered Ti-(8–18) at.% Nb alloys (0.8 wt.% oxygen in the alloys) [194].

Baker [192] firstly found that the SME in a Ti-35 wt.% Nb alloy, which originates from β → α″ MT, is alike the SME stems from the thermoelastic MT in other SMAs, such as NiTi, Cu-Zn-Al alloys. From then, many studies have been made about the effect of alloy content and thermo-mechanical treatment on microstructure evolution, MT temperature, SME/SE and mechanical behaviors [175,176,177,182,184,185]. However, only a few studies [193,194] present the exact phase transformation temperature for stress-free samples by common DSC methods, which had been attributed to small transformation enthalpy of β ↔ α″ MT and very low transformation temperature. Thus, most results of M_s_ temperature were obtained indirectly from the relationship between critical SIM stress and temperatures in stress-strain curves in Ni-free Ti-based SMAs [172,175,176,177]. Recently, Lai et al. found that the M_s_ temperature of Ti-Nb alloy can be measured by the DSC method at lower Nb composition, as shown in Figure 25 [194].

The microstructure evolution, including in-situ characterization [195,196], demonstrate that stable α phase, metastable α′ and ω phases often appear in Ti-based SMAs besides the primary β and α″ phases that are responsible for SME after rolling or heat treatment. However, the distribution and size of those phases would greatly influence the MT and mechanical behaviors. As is well known, ω phase is an important phase in Ti-based SMAs, which can be easily formed either by quenching from high temperature (athermal ω) or by aging at an intermediate temperature (isothermal ω) [197,198]. It had been reported that the precipitation of ω phase would cause embrittlement [199]. However, fine ω precipitates from β phase is an effective way to improve critical stress for slipping and SME, and stability of SE for Ti-based alloys [196,200]. It has been reported that, by annealing and proper aging treatment, nano-sized ω and α precipitates in β matrix of small grain size can further improve yield strength, fatigue life, and SME [201,202]. However, ω phase would coarsen or transform into α phase when aged at a higher temperature or longer duration [203], and SME or SE would deteriorate or even disappear. Fortunately, the formation or coarsening of ω phase can be inhibited by adding the third elements, such as Ta or Sn etc., in Ti-Nb binary alloys. Recently, Dubinskiy et al. [195] made in-situ X-ray diffraction (XRD) study during temperature changing or loading-unloading in Ti-Nb-Zr and Ti-Nb-Ta SMAs. They found only α″ phase forms for Ti-Nb-Ta SMAs during cooling, while the α″ and ω phase can be formed for Ti-Nb-Zr SMAs. Moreover, the quantity of α″ phase increases with cooling down or applying a load, while the quantity of ω phase is not affected.

**Figure 26:**
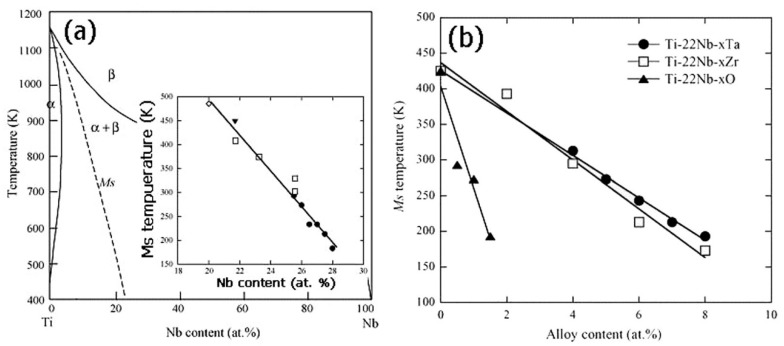
The effect of Nb on Ms (**a**) for Ti-Nb binary alloys and alloy elements on Ms (**b**) for Ti-Nb-based ternary alloys [172].

In addition to microstructure, the MT temperature of Ti-based SMAs is greatly affected by alloy compositions. Miyazaki et al. [172] reported that the M_s_ decreases by 40 K with 1 at.% increasing of Nb content, and the M_s_ is lower than RT when Nb content exceeds 25.5 at.% in binary Ti-Nb SMAs, as shown in Figure 26a. Furthermore, as shown in Figure 26b, the M_s_ temperature decreases by about 30, 35 and 160 K with 1 at.% Ta, Zr and O content for Ti-22at.%Nb-based ternary SMAs. Similarly, it is reported that the M_s_ decreases by 90 K [204], 160 K [205], and 200 K [206] with the addition of 1 at.% of Mo, Pt and N. In addition, Hao et al. found that M_s_ decreases 41.2 K and 40.9 K with increasing 1 wt.% Zr or 1 wt.% Sn, respectively, for Ti-(20–26)Nb-(2–8)Zr-(3.5–11.5)Sn alloys [207]. The change of M_s_ would lead to different SME and SE behavior of Ni-free Ti-based SMA. For example, Ti-(22–25)at.%Nb alloy exhibits only the SME at RT, while Ti-(25.5–27)at.% Nb alloys can show partial SME and partial SE [200].

**Figure 27:**
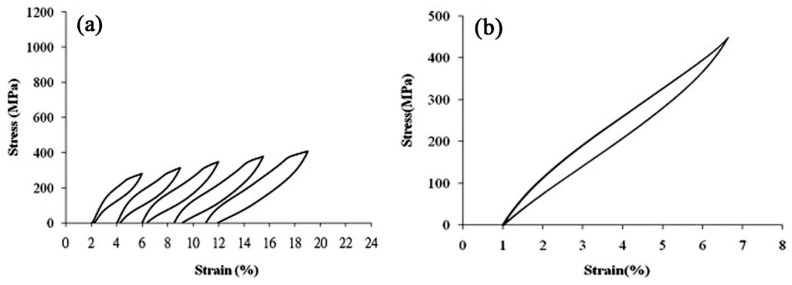
The stress-strain curves obtained by tensile test for Ti-19Nb-9Zr SMAs: (**a**) cyclic loading-unloading; (**b**) after five cycles of loading-unloading tests [209].

The SME and SE are also influenced by the critical stress for slip deformation in SMAs. Thus, low temperature annealing and aging treatment following cold-rolling were used to improve the critical stress of slip deformation by producing fine subgrain structure, fine α, and ω precipitates. Ti-Nb binary alloys can exhibit stable and almost complete superelasticity of 2.7% at RT [196]. In addition, the addition of third (or more) elements, such as Ta, Zr, O, Mo, Sn, etc., is also effective in promoting the critical stress for slip deformation and a larger transformations strain [203], and hence, improve the SE for Ti-based SMAs [208]. For example, by the addition of 4 at.% Mo, the maximum superelastic strain for Ti-15Nb-4Mo (at.%) can be increased to 3.5% at RT [203], as high as 4.3% recoverable strain for Ti-22Nb-6Zr (at.%) alloy at RT [178], which almost reach the possible maximum recoverable strain for those alloys at RT as revealed by in-situ XRD analysis [195]. Recently, a highest non-martensitic SE of 6% at RT, as shown in Figure 27, was reported in Ti-19Nb-14Zr (at.%) SMAs after annealing and aging treatment by Ma et al. [209], which is attributed to the presence of β and very fine ω phases.

**Figure 28:**
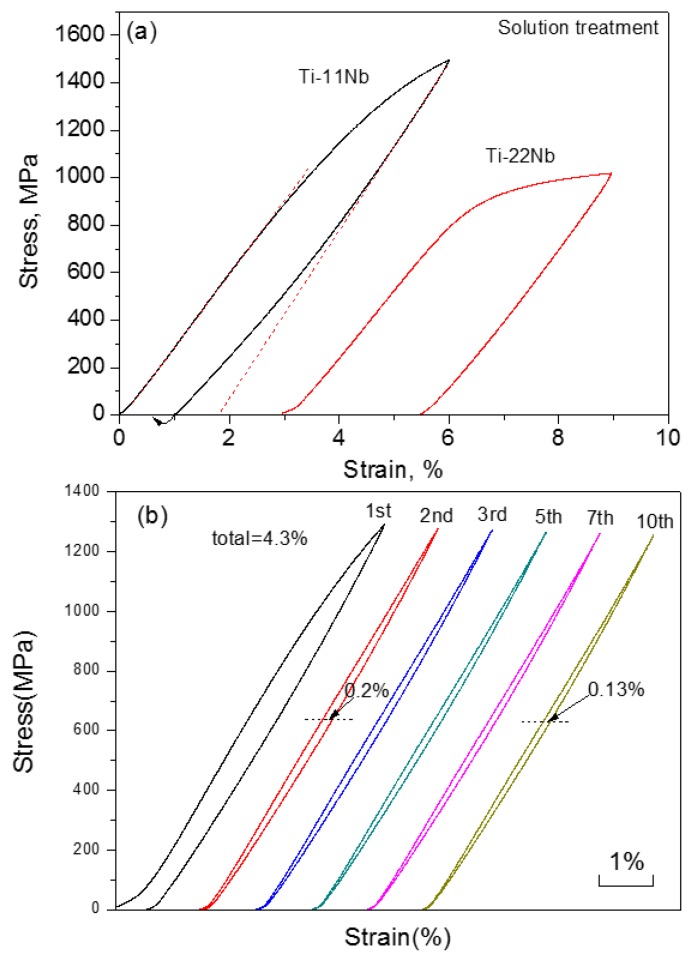
Compressive stress-strain curves of Ti-11Nb and Ti-22Nb alloys (**a**), and constant strain (pre-strain = 5%) cycling curves (**b**) of Ti-11Nb after solution treatment at RT [212].

Except for the melting method, the PM method, like HIP [210], was also used to produce Ti-based SMAs. Very recently, Lai et al. [211] fabricated Ti-22Nb-6Zr (at.%) SMAs with the porosity of about 5% by CS. The alloy exhibits as high as 5.9% compressive recoverable strain at −85 °C that is similar to that of as-rolled alloy, which is due to its very low M_s_ temperature. In order to increase the M_s_ temperature, Yuan et al. [212] decreased the Nb and Zr content to fabricated Ti-11Nb (at.%) alloys by CS, and 5.5% recoverable strain was obtained at RT, while Ti-22Nb (at.%) alloys show only 3.5% recoverable strain, as shown in Figure 28a. It is worth to note that the Ti-11Nb alloys can possess stable SE of 4.3% at RT after 10 cycles, as shown in Figure 28b. Table 3 lists some typical physical and SE properties of Ti-Nb-based SMAs.

#### 5.1.3. Mechanical and Biological Properties

In general, most of Ti alloys offer the appropriate mechanical properties for orthopaedic applications, but their susceptibility to crack propagation and relatively poor wear performance is unfavorable. Although dense Ni-free Ti-based SMAs exhibit the properties that are similar to β type Ti alloys, the composition and SE of Ni-free Ti-based SMAs are different from conventional β type Ti alloys. Thus, many studies have devoted to their mechanical behaviors, in particular, the effect of thermo-mechanical treatments or composition on the elastic modulus, ductility, or fatigue, etc.

**Figure 29:**
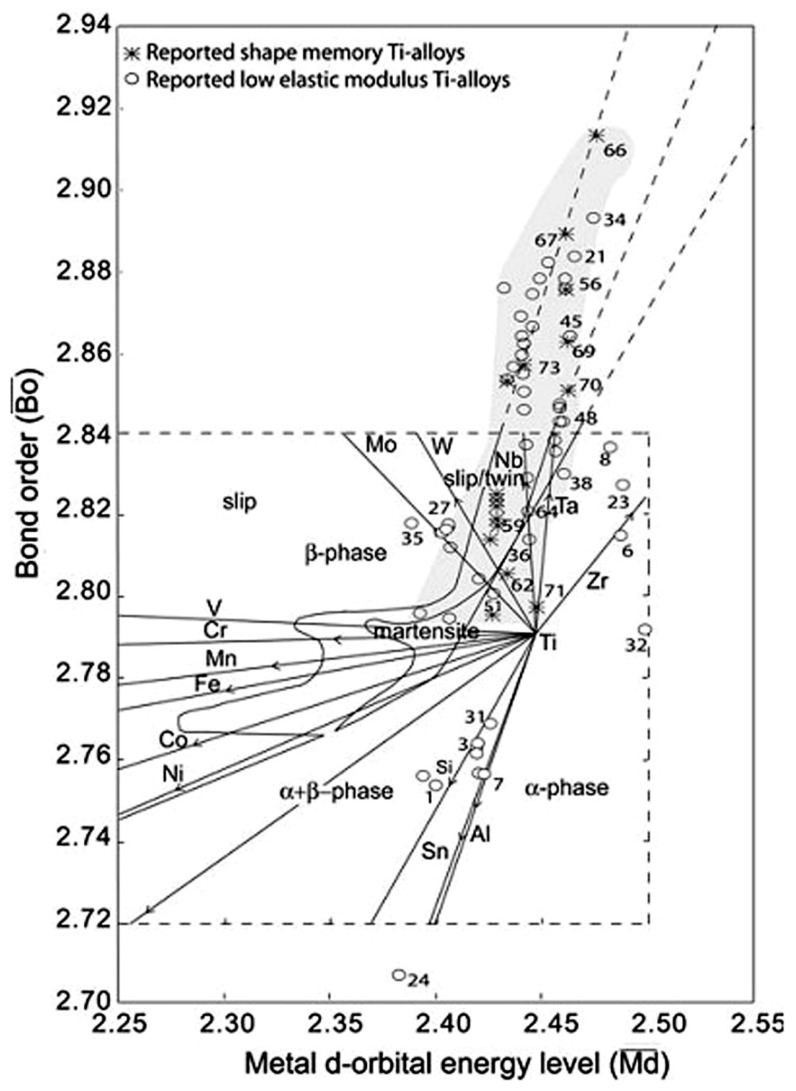
Extended phase stability index diagram based on $\bar{\mathrm{Bo}}$ and $\bar{\mathrm{Md}}$ parameters in which about 80 shape memory and low elastic Ti alloys. The shadow zone indicates the coexistence of both properties [216].

Ni-free Ti-based SMAs generally behave lower elastic modulus than α type Ti and α + β type Ti alloys because β phase is BCC crystal structure with lower atom density in the lattice. It has been reported that the elastic modulus can be tuned from 120 to 35 GPa by adjusting alloy composition, annealing and preferential crystal orientation [213], which approach the upper limit (25 GPa) of hard tissue. Moreover, the elastic modulus of Ti alloys can be approximately predicted by using the parameters that were proposal by Morignaga et al. [214] while using the following equations:

(2) $$\bar{\mathrm{Bo}}=\sum \mathrm{Xi}\left(\mathrm{Bo}\right)i$$

(3) $$\bar{\mathrm{Md}}=\sum \mathrm{Xi}\left(\mathrm{Md}\right)i$$

where X represents the atomic fraction, and M_d_ and B_o_ represent the values of the quantum parameters for each of the i alloying elements [215]. Each (Md, Bo) pair is located on the phase stability maps, as shown in Figure 29. The elastic modulus of the ternary alloys show strong $\bar{\mathrm{Md}}$ and $\bar{\mathrm{Bo}}$ dependence, and generally the alloy with high $\bar{\mathrm{Md}}$ and low $\bar{\mathrm{Bo}}$ has a low elastic modulus [216].

**Figure 30:**
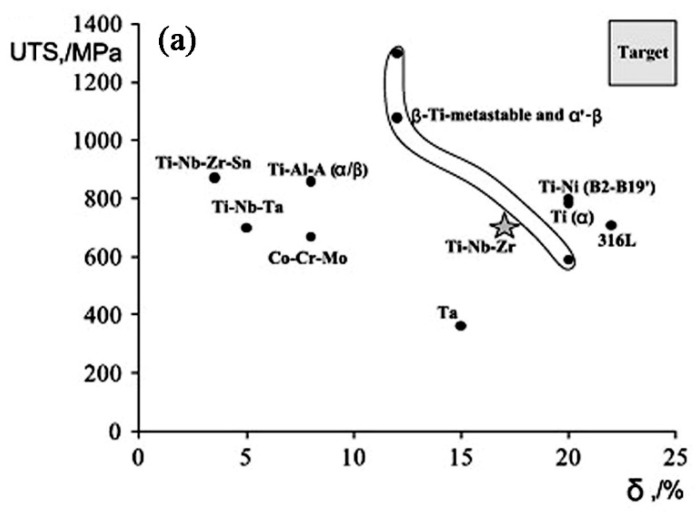

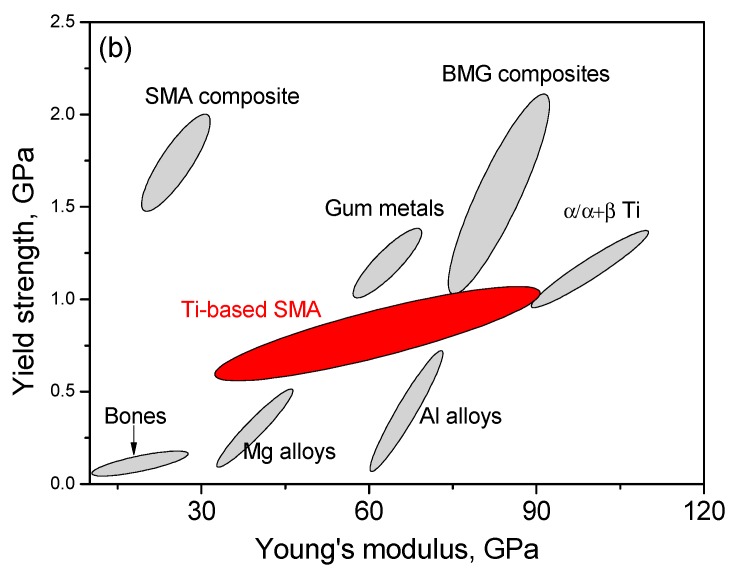
Mechanical properties of Ti-based SMAs, in comparison with other materials: (**a**) tension strength vs. elongation [217]; and, (**b**) yield strength v.s. young’s modulus.

Ni-free SMAs, including Ti-Nb-based [200] and Ti-Mo-based [185], show very good ductility, can easily cold-rolled to a thin plate of several mm thickness. Figure 30a compares the strength of Ti-based SMAs with other biomaterials [217], and they exhibit strength comparable to NiTi SMAs. Moreover, they combining high yield strength with low elastic modulus, as shown in Figure 30b.

**Figure 31:**
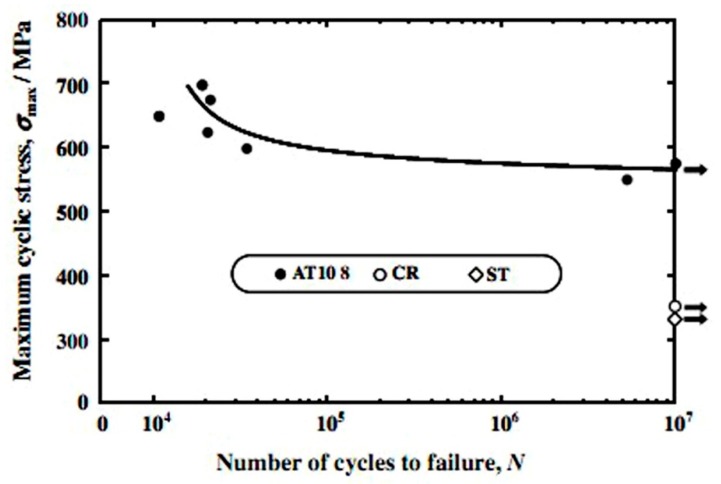
Fatigue properties of Ti-Nb-Ta-Zr alloys subjected to aging treatment at 573 K for 10.8 ks (AT10.8), solution treatment (ST), and severe cold rolling (CR) [223].

In addition to strength, the wear resistance of Ti-based SMAs should be a concern for hard-tissue replacement applications because the presence of wear debris in the surrounding tissue would cause loosening or failure of the implants. Because few investigations have been made on the wear performance of Ti-based SMAs, we summarized the wear performance of β type Ti-based bio-metals here. In general, pure Ti or Ti-6Al-4V alloys exhibit poor tribological performance due to their low resistance to plastic shearing and low protection induced by surface natural oxides [218]. Therefore, surface modifications or designing new Ni-free Ti alloys were used to ameliorate this problem. Fretting and sliding wear studies showed that Ti-35Nb-8Zr-5Ta (wt.%) is much superior to Ti-6Al-4V [219] due to SIM and high content of Nb_2_O_5_ formed in the surface layer [220]. Ehtemam-Haghighia et al. [221] reported that the β type Ti-11Nb-7Fe alloy can exhibit higher wear resistance and strength than pure Ti and Ti-6Al-4V alloys, as well as the lower elastic modulus. Thus, Ni-free Ti-Nb-based SMAs are believed to have good wear resistance when the applying stress is lower than the critical stress for slipping. Similarly, the fatigue performance of the solution treated β type Ti alloys can be improved by cold rolling [222] or appropriate aging treatment [223]. For example, the aged Ti-Nb-Ta-Zr alloys exhibit higher fatigue strength than that after solution treatment or severe cold rolling as shown in Figure 31, the fatigue life can reach as high as ~10^7^. Table 3 summarizes the mechanical parameters of Ti-Nb-based SMAs.

**Figure 32:**
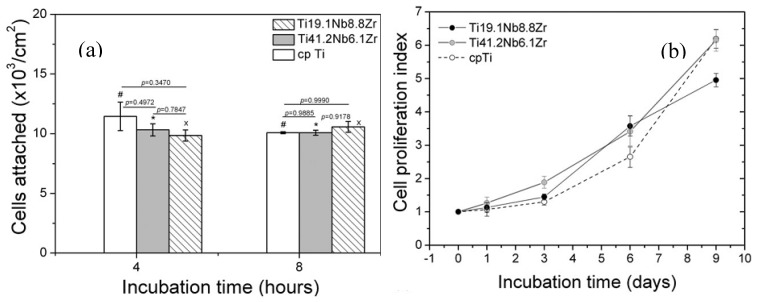
Attached number of living-MG63 cells (**a**) and cell proliferation index (**b**) as a function of the incubation time. The groups marked with the same symbol have no statistically significant differences at different times of culture [230].

**Figure 33:**
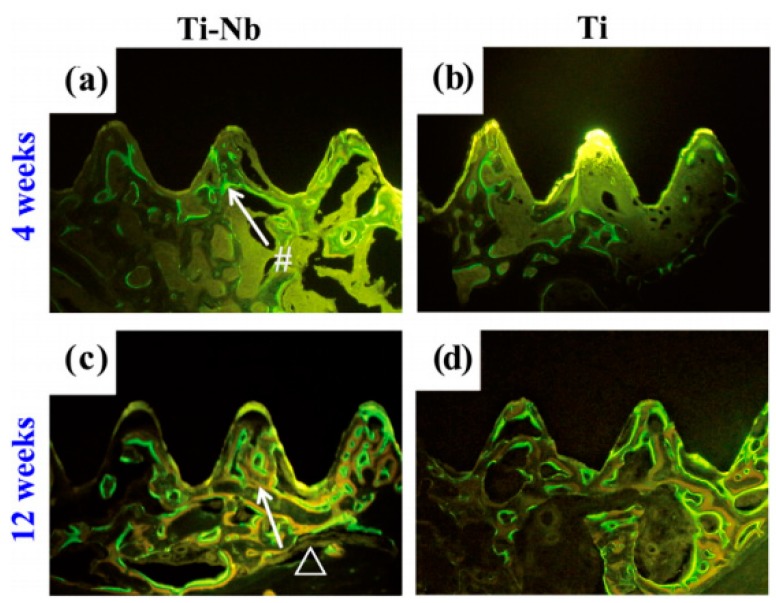
Histotomy of bone contact of the Ti–Nb alloy (**a**,**c**) and Ti (**b**,**d**) at 4 weeks and 12 weeks illustrated by fluorescence-dyeing reagents, respectively. Green (#) revealed the new bone formation of two-week duration dyed by calcein, and the yellow (Δ) revealed that new formation of four-week duration by tetracycline [231].

In general, pure Ti and Ti alloys possess excellent corrosion resistance and good biocompatibility under both in vivo and in vitro evaluation due to a passive TiO_2_ layer on their surface [224,225]. The addition of Nb and Ta can strengthen the surface TiO_2_ film of Ti alloys [226] and enhance the forming of highly stable Nb_2_O_5_ or Ta_2_O_5_ layer [227]. Thus, Ni-free β type Ti-based SMAs, often added with some non-toxic elements, such as Nb, Zr, Hf, and Ta, are supposed to exhibit good biocompatibility or low cytotoxicity in comparison to pure Ti. In fact, Ti-Nb-based (such as Ti-Nb-Zr) or Ti-Ta-based alloys had been proved to show better corrosion resistance than pure Ti or Ti-6Al-4V alloys in physiological or protein solutions [228], as well as excellent biocompatibility and osteoconductivity [194,229,230]. For example, Bai et al. reported that the Ti–Nb alloys exhibited corrosion resistance that was superior to Ti in three different physiological solutions [231], they found that the Ti–Nb alloys also produced no deleterious effect to L929 fibroblasts and human osteoblast-like MG-63 cells, and cells performed excellent cell attachment onto Ti–Nb surface, indicating a good in vitro cytocompatibility. In addition, Arciniegas et al. [230] carefully assessed the biocompatibility of two Ti-Nb-Zr SMAs by in vitro preosteoblastic cell testing, the results indicated that the cell biocompatibility is not statistically different to that obtained in pure Ti, as shown in Figure 32. Moreover, good cell adhesion is greatly attributed to the presence of a 2-nm thick layer of amorphous Nb_2_O_5_ on them. In addition, they also found that the Ti-19.1Nb-8.8Zr (wt.%) SMA exhibit higher corrosion resistance than NiTi SMAs [193], and even compared with pure Ti [232]. Bai et al. further compared in vivo bone tissue biocompatibility of Ti-Nb alloys with pure Ti, the results indicated that Ti-Nb alloys had a comparable osteocompatibility to pure Ti while using micro-CT and histological evaluations, as shown in Figure 33 [231].

Based on the above results on mechanical properties, superelastic behavior, corrosion resistance, and excellent biocompatibility, Ni-free Ti-based alloys could be a promising material for implantation.

### 5.2. Porous Ni-Free SMAs

As stated before, dense Ni-free SMAs exhibit low elastic modulus of ~35 GPa, which are much lower than pure Ti (110 GPa), and even smaller than that of NiTi SMAs (48 GPa). However, they are still much higher than hard tissues, for example, cortical bone (6–20 GPa) [30] and cancellous bone (<4 GPa) [31], and would produce a stress shielding effect and cause the failure of implantation. Inducing porous structure can reduce the elastic modulus. In addition, as mentioned before, the porous structure promotes cell adhesion, allows for bone cell in-growth and integrates with host tissue (i.e., osteointegration), as well as allowing body fluid exchange. Obviously, the porous structure is extremely important in determining the biological and mechanical properties without worrying about Ni ion from the high specific surface area, and consequently, the performance in orthopaedic applications. Accordingly, a number of efforts are made to fabricate and control the pore structure of Ni-free Ti-based SMAs in order to further promote their performances as hard-tissue replacements in recent ten years [210,217,233,234,235].

#### 5.2.1. Fabrication Method and Porous Structure

**Figure 34:**
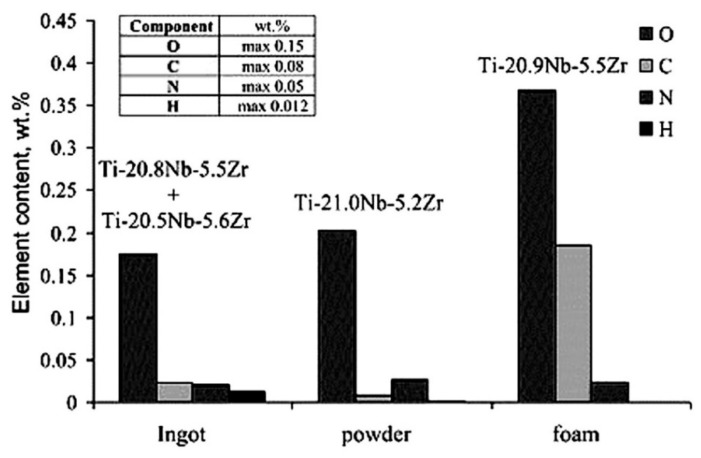
Oxygen, carbon, nitrogen, and hydrogen contents in bulk, powder and porous Ti-Nb-Zr specimens [210] (Inset contains maximum concentrations according to ASTM F67-00).

Distinct porous structures can be fabricated by different methods in metallic materials, such as porous Ti or Ta, or Al foams [47]. Because Ni-free SMAs consist of some elements with a high melting point, such as Ti (1660 °C), Nb (2468 °C), Zr (1850 °C), Ta (2995 °C), Mo (2617 °C), Hf (2150 °C), etc. Thus, most of porous Ni-free SMAs, are fabricated by PM or AM methods from element powders [233,234] or pre-alloy powder [217], including CS [210,217,235], microwave sintering [236], HIP/CF-HIP [237], and AM [238,239], which are similar to those for porous NiTi SMAs. However, SHS method cannot be adopted to preparing porous Ni-free SMAs due to no reaction between Ti and Nb (Mo or Ta). In addition, pore-forming agent, including NH_4_HCO_3_ [235,239,240], urea [241], TiH_2_ [237], or polyurethane [242,243], are usually added into the powders to obtain porous Ni-free SMAs with various high porosities and specific pore shape, because there is no obvious Kirkendall effect for Ti-Nb or Ti-Mo alloy system [211]. As like the fabrication of porous NiTi SMAs, as shown in Figure 34, Ni-free Ti-based SMAs also have high oxygen content and other impurities either dissolving in a matrix or forming oxides inside the matrix [210].

**Figure 35:**
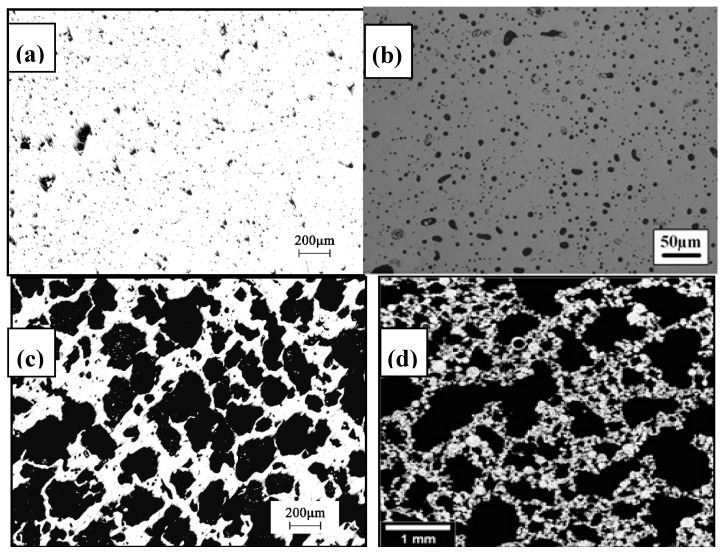
Pore morphology of porous Ti-22Nb-6Zr SMA with various porosities by different methods: (**a**) 6.7% by CS [241]; (**b**) 12% by CF-HIP [234]; (**c**) 57.6% by CS with NH_4_HCO_3_ [241]; and, (**d**) 65% by CS with pore forming agent from alloy powder [217].

**Figure 36:**
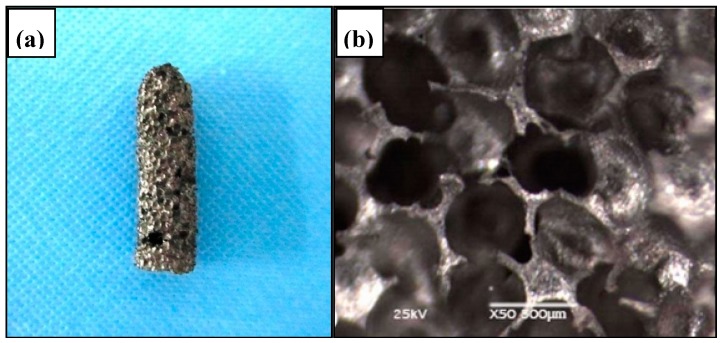
Porous Ti-25 wt.% Nb alloys with 70% porosity fabricated by PM using polyurethane as space holder [243]: (**a**) macrographic image, and (**b**) pore structure.

For Ni-free Ti-Nb-based SMAs, as shown in Figure 35a,b, it is easy to produce porous specimens with low porosity (5–6%) and very small pore size (<10 μm) [241] by sintering at high temperature (1400 °C), while it is difficult for Ni-Ti alloy system. In order to obtain high porosity, the pore-forming agent [235,240,241,242] has to be added into the mixed element powders to form pores. Figure 35c is the morphology of porous Ti-22Nb-6Zr alloys that were prepared by CS with NH_4_HCO_3_ addition [241], and it has 58% porosity and near spherical pores of ~250 μm. By controlling pore forming agent, the larger pore size of 800–1000 μm and high open porosity can be obtained by vacuum sintering from alloy powders, as shown in Figure 35d. Xu et al. fabricated porous Ti-25 wt.% Nb alloys with porosity even reaching 70% by sintering using polyurethane as space-holder, as shown in Figure 36 [243]. In addition, the pores shape of porous Ni-free SMAs is mainly determined by the particle shape of the pore-forming agent and it can be easily adjusted [232].

Except for the pore structure, it is vital to obtain β phase as a primary phase and homogeneous microstructure for porous Ni-free Ti-based SMAs fabricated by PM. However, the Ti solid solution of porous Ti-Nb(or Mo)-based SMAs only formed through inter-diffusion of Ti and Nb (Zr and Ta) atoms. Therefore, the homogeneous single phase can only be obtained by sintering at a higher temperature (>1400 °C) for long duration (10 h) [233,241]. For example, porous Ti-22Nb-6Zr (at.%) alloys sintered at 1200 °C for 10 h contains more undesirable phases than that sintered at 1400 °C for the same time, such as α-Ti, Nb, and Zr. While single β phase, can be obtained at the higher sintering temperature.

**Figure 37:**
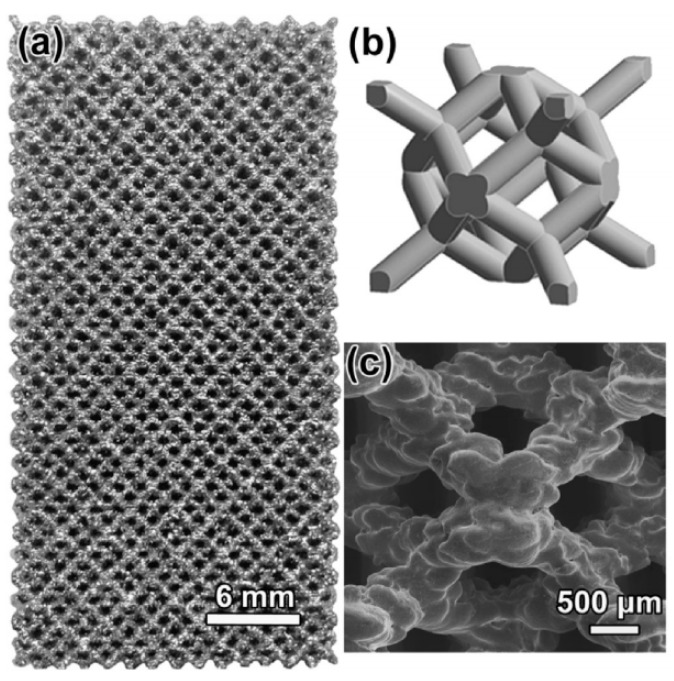
(**a**) The morphology of the electron beam melting (EBM)-produced porous Ti-24Nb-4Zr-8Sn (wt.%) SMAs; (**b**) the single unit of 3D rhombic dodecahedron modeling; and, (**c**) the surface morphology [239].

Porous Ni-free SMAs have been also fabricated by AM methods using pre-alloyed powders in recent years, because they have the ability to build complex porous structure according to the CAD model. For example, Liu et al. [239] prepared porous Ti-24Nb-4Zr-8Sn (wt.%) SMAs with a single β phase by electron beam melting (EBM) technique, as shown in Figure 37a. The porous architecture can be exactly obtained according to the designed model, as shown in Figure 37b,c. Moreover, the high porosity of 68–91%, pore size of 500 μm, and 3% SE can be achieved in these porous samples.

In summary, the porous structure of Ni-free SMAs can be adjusted to satisfy the bone replacement requirements (pore size of 100–500 μm, and porosity of 30–90%) by adding a pore-forming agent during PM or by AM techniques. Moreover, the single and homogeneous microstructure can also be obtained through the optimal processing parameters.

#### 5.2.2. Mechanical and Biomedical Properties

The major benefits of porous NiTi SMAs as compared with other bone graft materials are their good mechanical strength, low elastic modulus, and high recoverable strain at body temperature [65]. Therefore, it is necessary for porous Ni-free SMAs to match these benefits.

The mechanical properties of porous Ni-free SMAs are also greatly affected by pore characters, such as porosity or pore shape. The mechanical properties of porous Ni-free SMAs deteriorate to a very low level with increasing porosity and pore size. Thus, a trade-off must be maintained between the mechanical properties and porosity (or pore size) for different biomedical applications [244]. However, most of the studies [210,217,236] focus on the dependence of the elastic modulus and compressive strength on porosity, but not on bending or tensile. Moreover, few investigations reported the relationship between SE, fatigue, or wear properties and porosity for porous Ni-free SMAs.

**Figure 38:**
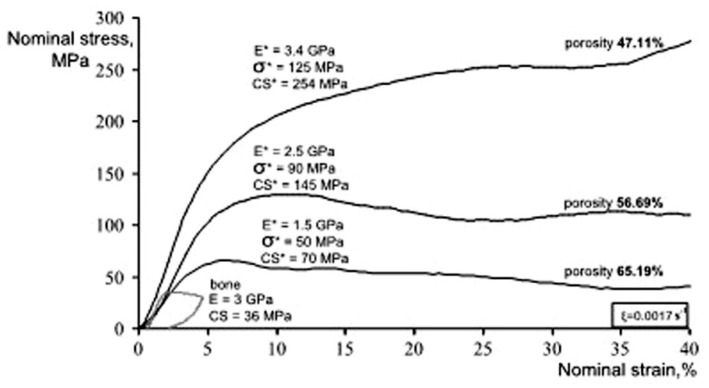
Compressive stress-strain curves of porous Ti-Nb-Zr alloys and cortical bone [210].

**Figure 39:**
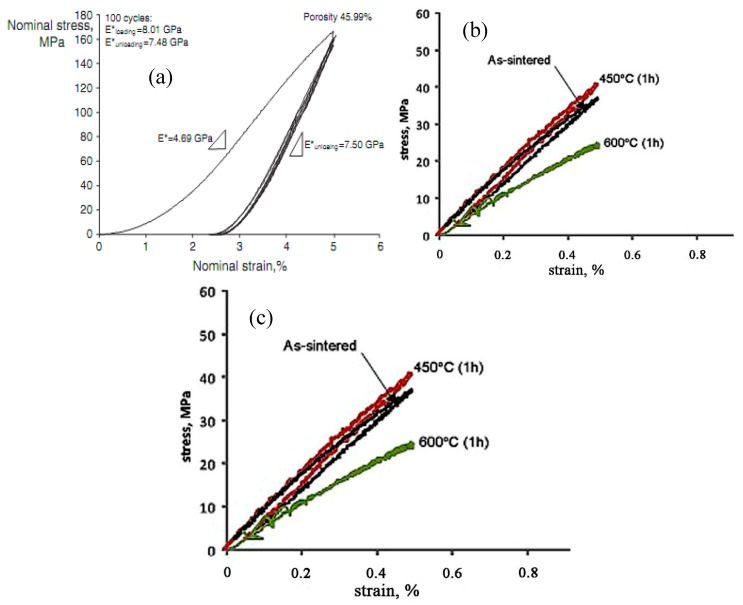
Stress-strain cycle curves for porous Ti-21Nb-5.5Zr alloys under various testing modes at RT: (**a**) compressive; (**b**) tensile; and, (**c**) bending [210].

Porous Ni-free SMAs can exhibit a high compressive elongation of more than 40%, and their compressive strength (70–125 MPa) and elastic modulus (1.5–3.4 GPa), which decrease with an increasing of porosity in the range from 47% to 65%. Moreover, the superelastic strain of these porous Ti-Nb-Zr SMAs at RT is similar to that of cortical bone, being 2–2.5%, as shown in Figure 38. These make the porous Ni-free SMAs match the properties of bovine trabecular bone [210]. However, the SIM plateau cannot be observed for all the porous Ni-free SMAs [214,221,234,235]. On the contrary, they usually exhibit linear superelastic behavior during either compressive [210,217,243], tensile or bending testing [210], as shown in Figure 39. It is obvious that porous Ni-free SMAs show higher compressive strength and SE (3.5% recoverable strain), than that from tensile or bending, and the worst results appeared in tension testing (0.5% recoverable strain). The reason is that the cracks easily propagated from the tip around pores during tension.

**Figure 40:**
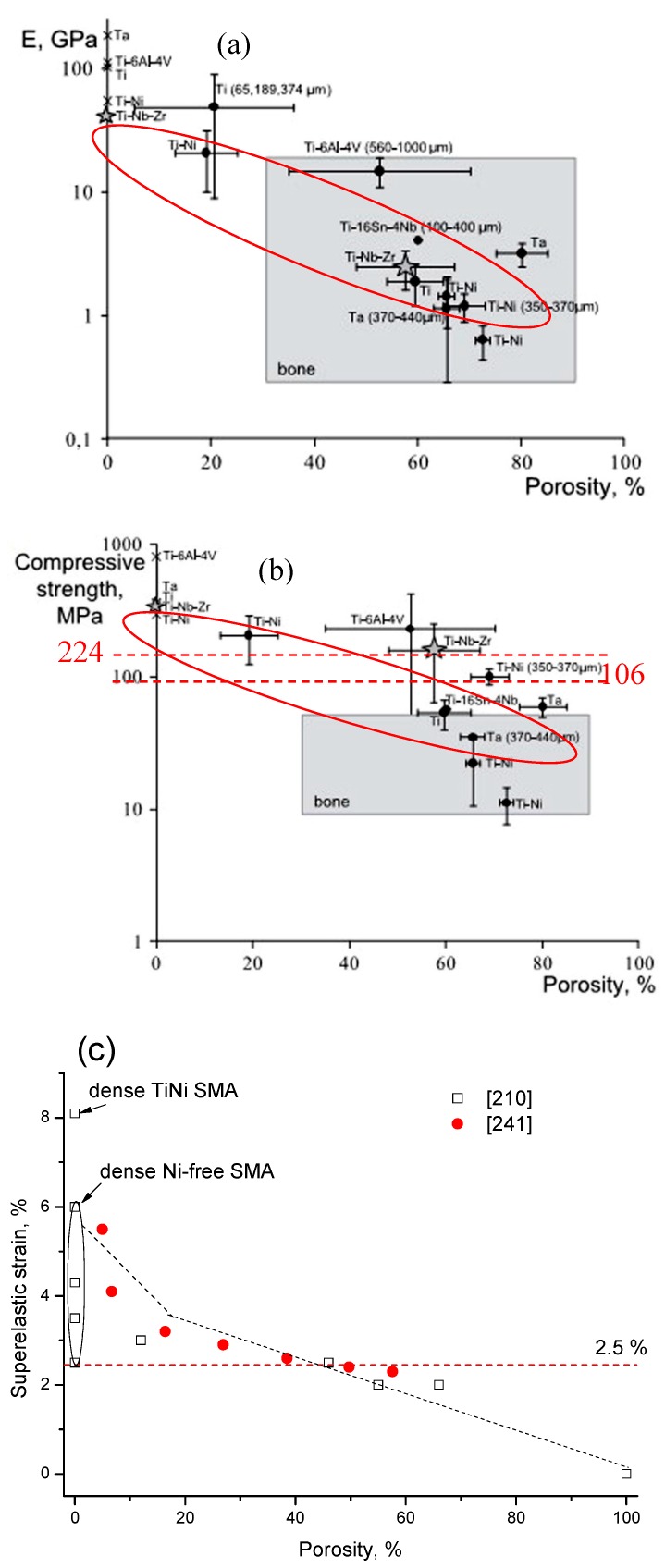
The relationship between elastic modulus (**a**); compressive strength; (**b**) [210]; and, superelastic strain (**c**) [210,217,241] and porosity for Ni-free SMAs.

Generally, the dependence of elastic modulus and compressive strength on porosity can be roughly predicted using Gibson and Ashby model [48] for porous Ni-free SMAs [235]. However, it should be noted that the mechanical behavior of porous alloys is also affected by the pore size, shape and distribution, in addition to porosity [245,246]. The elastic modulus can be adjusted from 35–100 GPa (dense Ni-free SMAs) to 1–2 GPa (60–70% porosity). The relationship map of elastic modulus and porosity for porous Ni-free SMA are summarized in Figure 40a [210], referring to Figure 16a for comparison with porous NiTi SMAs. Thus, it is clear that porous Ni-free SMAs can also show a competitive combination of “elastic modulus–porosity” properties and they can match perfectly that of human bone [210] when compared to the known porous NiTi and porous metallic materials, such as Ta, Ti, etc.

Moreover, from the “compressive strength–porosity” point of view, as shown in Figure 40b, porous Ni-free SMAs can exhibit the “strength-porosity” region higher than that of human bone, and it is similar to porous NiTi SMAs shown in Figure 16b. Thus, the safe use of them is well guaranteed as biomedical implantation materials [210]. The recoverable strain is another important property that should be taken into account for porous Ni-free SMAs. There are a few studies focus on the relationship of superelastic strain and porosity. Figure 40c summarizes those reported data [210,217,241]. Obviously, the changing trend of them is different from that of porous NiTi SMAs in Figure 16c, it decreases rapidly from dense to 20% porosity, and the slope of reducing becomes mild, from 20% to 65% porosity. It is clear that porous Ni-free SMAs (<45% porosity) can show maximum recoverable strain higher than cortical bone (2.5%), though it exhibits slight lower superelastic strain than porous NiTi SMAs at various porosities. Nevertheless, porous Ni-free SMAs are still superior to other metallic foams.

**Figure 41:**
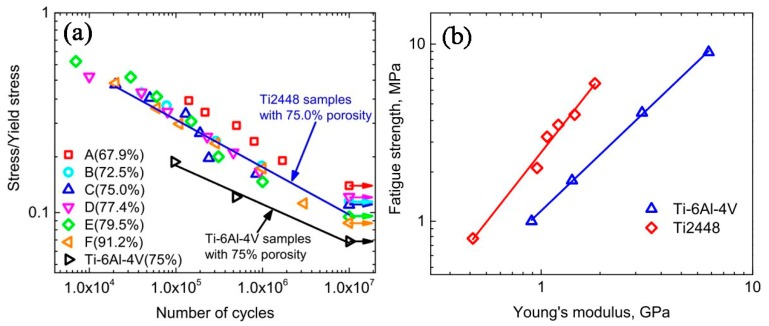
(**a**) The normalized S-N curves of the porous Ti2448 and Ti-6Al-4V alloys with different porosities, and (**b**) the relationship of Young’s modulus and the fatigue strength for the porous Ti2448 and Ti-6Al-4V specimens [239].

For the hard tissue replacement applications, the fatigue properties of porous Ni-free SMAs are one vital aspect to be considered due to experiencing cyclic loading during daily activities. In general, the fatigue properties of highly porous Ti alloys may suffer from high levels of porosity, and the fatigue strength of porous Ti alloys has been reported to be in the range of 0.1–0.25 yield strength of porous samples, which is lower than the normalized endurance limit of dense titanium (i.e., 0.4 yield strength). The rough surface of struts, notch sensitivity of Ti alloys, the presence of void and porosity in struts, microstructure, and residual stress are believed to be the cause of this difference [247]. Leuders et al. [248] found that heat treatment and the HIP process can significantly increase the endurance limit of porous Ti alloys fabricated by AM. Recently, Liu et al. [239] fabricated porous Ti-24Nb-4Zr-8Sn (Ti2448) alloys with EBM and the following annealing treatment, they found that porous Ti2448 alloys with SE can exhibit a higher normalized fatigue strength (Figure 41a), greater plastic zone ahead of the fatigue crack tip, and the crack deflection behavior in comparison with porous Ti-6Al-4V alloys. Moreover, for the same fatigue strength, the Young’s modulus of porous Ti2448 samples is only half of the porous Ti-6Al-4V samples, as shown in Figure 41b [239]. Thus, porous Ni-free Ti-based SMAs can exhibit better fatigue performance than porous pure Ti or Ti alloys.

It has been proved from many results that porous Ni-free Ti-based SMAs exhibit excellent corrosion resistance [226,227,228] and bio-inert to human body [249]. They are suitable for biomedical applications especial for bone replacement after treating by bioactive surface modification [250,251]. For example, Li et al. [252] reported analogically that porous Ti-24Nb-4Zr SMAs owns excellent corrosion resistance at 0.9% physiological and Flank’s solutions with different pH values at body temperature. Moreover, Ti-Nb-Sn SMAs exhibit even better corrosion resistance than NiTi SMAs [253], and there are no any harmful ions releasing from them [237]. In addition, the bioactive HA layer can form on the inner and outer surfaces of porous Ti-Nb-Sn [237] and Ti-Mo [254] SMAs modified by alkali-heat treatment. Similar to other porous SMAs, the rough inner surfaces of porous Ni-free SMAs exhibit better apatite-inducing ability and cell growth than the smooth surface of dense materials, and their high specific surface area is more favorable for cell adhesion and proliferation [253]. For instance, the porous Ti-Nb-Zr alloys show three times cell numbers than that on the dense samples [14].

**Figure 42:**
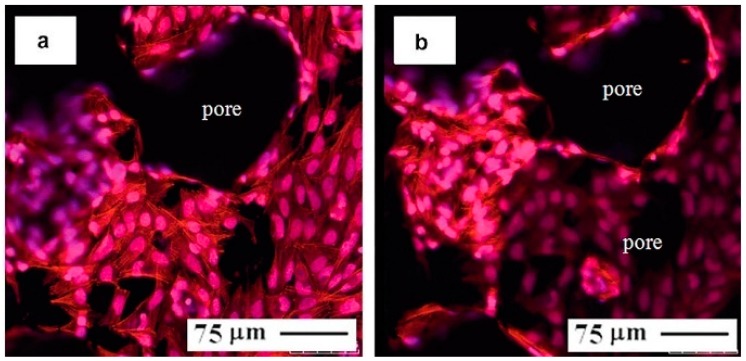
Confocal micrographs of cell growth: (**a**) inside pores and (**b**) on the surface of the porous TiNbZr alloys [14].

Most of the reported studies in vitro indicated that porous Ni-free SMAs exhibit good cell biocompatibility, as shown in Figure 42, which is significant for implantation or replacement materials. However, the detailed in vivo results are still lack about cellular metabolism, gene expression, and blood compatibility, as well as the effect of pore structure on biocompatibility. Here, we just presented some results of the effect of the pore on biocompatibility from porous Ti alloys, which can indicate the porous Ni-free Ti-based SMAs indirectly.

With respect to the porous Ti alloys, the optimal pore size of 100–500 μm had been accepted widely for bone replacements to allow bone cell in-growth [255,256]. Moreover, Clemow et al. [257] reported that the percentage of bone growing into the surface was inversely proportional to the square root of pore size. In addition to pore size, the pore shape would also affect the extent of cell in-growth. Goodman et al. [258] had reported that the bone in-growth in square-shaped pores increases in comparison with that in round-shaped pores. Pores with more ragged and rough surfaces also offer a larger surface area for bone in-growth [259]. Tuchinskiy et al. [260] selected porous Ti alloys with different porosities and implanted them into mice for four weeks. The results indicated that the specimen with low porosity provoked a more vigorous foreign body reaction, and was encapsulated in a dense, highly collagenous bag with few blood vessels running through it, while the material with high porosity had a thinner sac with far greater vascularity.

**Figure 43:**
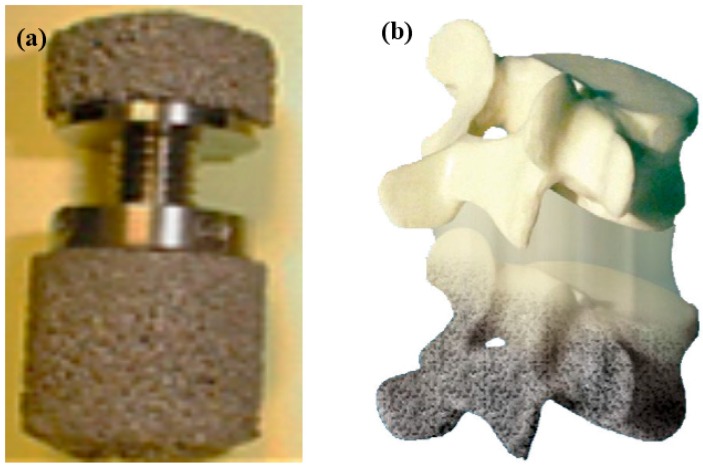
Biomedical applications made by porous Ni-free SMAs: (**a**) spine implantations [167]; and, (**b**) spine complete replacements [263].

In summary, the mechanical properties of porous Ni-free SMAs, especially with the elastic modulus and compressive strength as the two key factors, can be tuned to match various bone replacement applications, such as cancellous bone (<4 GPa), cortical bone (6–20 GPa), etc. Its SE at body temperature can also reach 2.5% when the porosity is smaller than 40%, and it can be improved further by optimizing pore architecture and microstructure. Besides, porous Ni-free alloys possess biocompatibility that is as good as porous Ti. Thus, porous Ni-free SMAs possess suitable mechanical and biomechanical properties required for hard-tissue replacement applications proposed in Section 3, while the worry for Ni ion releasing no longer exists. Thus, these make them competitive materials (even superior to others porous pure Ti or porous Ti-6Al-4V alloys [261]) for hard-tissue implantation, such as various biological fixation applications [262], or for biomedical scaffolds in tissue engineering applications [263], as shown in Figure 43. All of the biomedical application examples that are discussed in Section 4.4 are also applicable to porous Ni-free SMAs. Moreover, porous Ni-free SMAs are more suitable for the long-term hard-tissue replacements, such as spine complete replacement materials, as shown in Figure 43b.

## 6. Prospects and Summaries

Porous SMAs, including porous NiTi SMAs and Ni-free SMAs, can be fabricated by PM combing with space-holder or AM techniques to possess various complex porous architectures mimicking the microstructure of different hard-tissue parts with excellent flexibility and reproducibility. Moreover, external figuration can also be easily obtained to promote individualized implant design. This opens a pathway to a wide range of potential hard-tissue replacement applications. In addition, the control of porosity in matrix struts, pore surface roughness, and phase distribution is essential to reduce stress concentration and corrosion, which can greatly improve their fatigue failure, wear, and corrosion performance, thus enhancing long-term reliability.

Both NiTi SMAs and Ni-free SMAs can provide similar modulus with enhanced strength and fatigue life matching between the replacement materials and the hard-tissues. Moreover, they possess superior shape recovery capability to match the hard-tissue. Thus, they can fulfill almost all of the structural and property requirements for various hard-tissue replacement applications. In addition, some valid numerical (or finite element) modeling techniques are capable of establishing the mechanical and biological, as well as superelastic and biomechanical behavior that is dependent on the porosity of these porous SMAs. With continuous amelioration of those methods, a more actual relationship will be provided between the performance and fine pore structure, as well as attractive alternatives for lengthy experimental evaluation, such as fatigue testing. It will promote the new design for hard-tissue replacement applications.

Porous NiTi SMAs after surface modification exhibit good biocompatibility and acceptable Ni leaching level, while porous Ni-free SMAs show excellent biocompatibility without any harmful ion releasing. Furthermore, some extra efficiency-related improvements, such as bioactive layer (e.g., HA protective film), on the pore surfaces to enhance the functionality of bone in-growth, wear resistance, and antibacterial character, can be also implemented. In order to improve the long-term reliability, hybrid coating (such as bioactive nanofiber hybrids coating) and in-situ surface modification should be the future trend for surface modification of porous SMAs.

Consequently, porous SMA is considered one of most competitive candidate for hard-tissue replacement materials. Porous NiTi SMAs after surface modification are suitable for relatively short-term applications (e.g., the implants are used in the patients of more than 80 years old and they usually exist in the human body for less than 10 years) under high and complex loading conditions according to the current experimental results. However, the long-term applications should be further evaluated depending on more evidence. While porous Ni-free Ti-based SMAs seem suitable to apply under relatively low loading condition for a longer period (more than 10 years, e.g., adopting in the younger patients of aged 60–79) until now. We believe that porous Ni-free SMAs will be the most competitive candidate for hard-tissue replacement applications by further improving their mechanical and superelastic properties.

Nevertheless, the human body is a very complex organic system, which may cause an unpredictable adverse reaction when the devices with some unsolved problems are implanted into it. There are several aspects need to be further studied.

The first, the porous NiTi and Ni-free Ti-based SMAs are mainly prepared by PM methods, which would easily cause high oxygen content (or another impurity) in materials by dissolving in the matrix or forming oxides inside the matrix [210]. Moreover, it is well known that the properties of Ti and its alloys are very sensitive to interstitial elements, such as O, N, C, and H [264]. The M_s_ temperature would reduce to very low due to the presence of O. Thus, porous NiTi and Ni-free SMAs behave totally different mechanical or superelastic behaviors to dense forms at the same nominal composition. Moreover, the secondary phases induced due to high content interstitial elements would greatly deteriorate the fatigue and corrosion properties for porous Ni-free and NiTi SMAs. Thus, the contents of these interstitial elements, especially oxygen, must be controlled to a very low level, and it seems an effective method to prepare powder and sintering under a reductive atmosphere.

The second, for some bone replacements, such as total hip replacements, the properties of tensile, bending, fatigue-corrosion, and wear-corrosion must be important in addition to compressive strength and elastic modulus. Indeed, it is known that the tensile cyclic strain has been shown to affect the morphology, directionality, and proliferation of soft tissue cells [265,266], and the biological activity of bone cells in vitro [267]. However, most of the reported results come from compressive testing for porous SMAs. Thus, tensile or bending should be extensively studied for porous NiTi and Ni-free SMAs.

The third, although a rough “property-porosity” map has been built up for porous NiTi and Ni-free SMAs, it is still difficult to predict exactly all of the properties based on its specific pore structure. Moreover, except porosity, pore size and pore shape would also affect the performances. Thus, a precision relationship map should be built up between the properties and pore structures by future work.

Finally, the interaction between porous SMAs with tissue cells in the field of tissue engineering is still unknown, and the study on how efficiently to evaluate this network performing as a part of the circulatory system needs to be strengthened in the future.

## Figures and Tables

**Table 1:** Physical and mechanical properties of human bone and teeth [22,23,24,25,26,27,28,29,30,31,32,33,34,35].

	Hard-Tissue	Human Bone		Human Teeth	
Properties		Cortical Bone	Cancellous Bones	Enamel	Dentin
density		1.99 g/cm^3^ [28]	0.05–1 g/cm^3^ [29]	3 g/cm^3^	2–2.1 g/cm^3^
porosity		5–30% [22,24,25,26,27]	30–90% [22,29]	7%	59–70%
Pore size		10–500 μm [22,23,24,25,26,27]	50–300 μm [24,25,26,27]		1.7–2.5 μm [33]
Tension Strength		79–151 MPa(longitudinal) [28] 51–56 MPa (transverse)			34–61 MPa [34]
Compression strength		131–224 MPa(longitudinal) 106–133 MPa(transverse) [28]	2–5 MPa [31]		
Elastic modulus		17–20 GPa(longitudinal) [30,31] 6–13 GPa(transverse)	0.76–4 GPa [31]		3–25 GPa [35]
Recoverable strain		2–2.5% [32]			

**Table 2:** Pore characteristics of porous NiTi SMAs by various techniques [51,52,53,54,55,56,57,58,59,61,63,64].

Fabrication Method	Porosity/%	Pore Size/μm	Pore Shape	Pore Connectivity	Pore Distribution
CS	20–50%	10–100	irregular	good	homogeneous
HIP/CF-HIP	10–80%	100–3000	rounded	bad	homogeneous or gradient
SHS	30–70%	300–500	directional	very good	homogeneous
Space holder assisted sintering	10–80%	10–3000	Depend on space holder	Depend on the space holder	homogeneous or gradient
AM	0–90%	>150	Depend on designing	very good	homogeneous or gradient

**Table 3:** Typical properties for Ti-Nb-based SMAs [40,198].

**Melting point**	1750–1800 °C	**Elastic Modulus**	35–120 GPa
**Density**	4.8–5.5 g/cm^3^	**Poisson’s Ratio**	0.33
**Yield Strength**	250–790 MPa	**Tension strength**	590–1074 MPa (rolling and aging)
**Biocompatibility**	Excellent, compared Ti	**Elongation**	15–90%
**Corrosion resistance**	Excellent, better than Ti	**Recoverable strain**	Maximum 6%
**Wear resistance**	Good, better than Ti	**Fatigue life**	3 × 10^4^–1 × 10^7^
